# Frequency-Specific Changes in the Fractional Amplitude of the Low-Frequency Fluctuations in the Default Mode Network in Medication-Free Patients With Bipolar II Depression: A Longitudinal Functional MRI Study

**DOI:** 10.3389/fpsyt.2020.574819

**Published:** 2021-01-08

**Authors:** Jun Zhou, Xiaoqian Ma, Chunwang Li, Aijun Liao, Zihao Yang, Honghong Ren, Jinsong Tang, Jinguang Li, Zongchang Li, Ying He, Xiaogang Chen

**Affiliations:** ^1^Department of Psychiatry, The Second Xiangya Hospital, Central South University, Changsha, China; ^2^National Clinical Research Center for Mental Disorders, Changsha, China; ^3^National Technology Institute on Mental Disorders, Changsha, China; ^4^Hunan Key Laboratory of Psychiatry and Mental Health, Changsha, China; ^5^Mental Health Institute of Central South University, Changsha, China; ^6^Department of Radiology, Hunan Children's Hospital, Changsha, China; ^7^Department of Psychiatry, Sir Run Run Shaw Hospital, Zhejiang University, Hangzhou, China

**Keywords:** fractional amplitude of low-frequency fluctuations, bipolar depression, resting state functional magnetic resonance imaging, default-mode Network, escitalopram, lithium

## Abstract

**Objective:** This study aimed to examine the treatment-related changes of the fractional amplitude of low-frequency fluctuations (fALFF) in the default mode network (DMN) across different bands after the medication-free patients with bipolar II depression received a 16-week treatment of escitalopram and lithium.

**Methods:** A total of 23 medication-free patients with bipolar II depression and 29 healthy controls (HCs) were recruited. We evaluated the fALFF values of slow 4 (0.027–0.073 Hz) band and slow 5 (0.01–0.027 Hz) band of the patients and compared the results with those of the 29 HCs at baseline. After 16-week treatment of escitalopram with lithium, the slow 4 and slow 5 fALFF values of the patients were assessed and compared with the baselines of patients and HCs. The depressive symptoms of bipolar II depression in patients were assessed with a 17-item Hamilton Depression Rating Scale (HDRS) before and after treatment.

**Results:** Treatment-related effects showed increased slow 5 fALFF in cluster D (bilateral medial superior frontal gyrus, bilateral superior frontal gyrus, right middle frontal gyrus, and bilateral anterior cingulate), cluster E (bilateral precuneus/posterior cingulate, left cuneus), and cluster F (left angular, left middle temporal gyrus, left superior temporal gyrus, and left supramarginal gyrus) in comparison with the baseline of the patients. Moreover, a positive association was found between the increase in slow 5 fALFF values (follow-up value minus the baseline values) in cluster D and the decrease in HDRS scores (baseline HDRS scores minus follow-up HDRS scores) at follow-up, and the same association between the increase in slow 5 fALFF values and the decrease in HDRS scores was found in cluster E.

**Conclusions:** The study reveals that the hypoactivity of slow 5 fALFF in the DMN is related to depression symptoms and might be corrected by the administration of escitalopram with lithium, implying that slow 5 fALFF of the DMN plays a key role in bipolar depression.

## Introduction

Depression is the most common manifestation of mood state in bipolar disorder II, which is responsible for high suicide rates (1) and significant functional impairment (2). The treatment effect of bipolar depression is closely related to its prognosis. Medication therapy is the most common treatment for bipolar II depression, and although the functional and structural abnormalities of this disease are revealed by a large number of MRI results (3, 4), how the brain can be modulated by medication therapy and the corresponding changes from the neuroimaging perspective remains unclear.

The amplitude of low-frequency fluctuations (ALFF) is an index of local spontaneous neural activity in the resting state (5, 6) and reflects regional energy metabolism and activity of chemical synaptic signaling in the brain. The ratio of the power spectrum of low frequency to that of the entire frequency range is defined as the fractional amplitude of low-frequency fluctuations (fALFF). Compared with ALFF, fALFF can suppress non-specific signal components in fMRI and is more sensitive and specific to the detection of spontaneous brain activities (7). Recently, fALFF has been used in investigating neurobiological mechanisms and treatment effects in various psychiatric disorders [e.g., schizophrenia (8) and panic disorder (9)]. Several cross-sectional whole-brain fALFF studies on bipolar depression have suggested that when compared with healthy controls (HCs), patients showed aberrant fALFF in the superior frontal gyrus (SFG) (10, 11) and precuneus (PCu) (12), which belong to the default mode network (DMN). Treatment-related changes of escitalopram in fALFF were observed in patients with panic disorder (9) and major depression (13), and the changed brain region, including the medial prefrontal cortex (mPFC), was located in the DMN. However, treatment-related changes of fALFF within the DMN on bipolar II depression still remain elusive.

Although low-frequency oscillations (LFO) ranging from 0.01 to 0.25 Hz are physiologically relevant and related to neuronal fluctuations, researchers found that LFOs with a range of 0.01–0.073 Hz (slow 4: 0.027–0.073 Hz, slow 5: 0.01–0.027 Hz) often embody the spontaneous activities of neurons in the gray matter and LFOs with a range of 0.073–0.025 Hz (slow 3: 0.073–0.198 Hz; slow 2: 0.198–0.25 Hz) are often detected within the white matter (14, 15). Thus, fALFF with 0.01–0.08 Hz frequencies is often explored in the gray matter study of mental illness (16, 17). Recent research further reported that the cortex and subcortical nuclei signal different bands in slow 4 and slow 5. Slow 4 is the most robust in the subcortical nuclei, such as the basal ganglia, and the strongest signal of slow 5 is found in the cortex, such as the mPFC in healthy people (15). Based on these findings, slow 5 and slow 4 fALFF have been used in exploring some psychiatric diseases, such as mild cognitive impairment with mild depression (18), chronic primary insomnia (19), and post-stroke depression (20) to improve precision. One cross-sectional study displayed different results in slow 5 and slow 4 fALFF in psychotic bipolar disorder (21), suggesting that the medication-related changes in fALFF in bipolar II depression may also lead to different results in slow 4 and slow 5.

The DMN consists of the mPFC, PCC/PCu, inferior parietal lobule (IPL), and lateral temporal cortex (LTC) (22, 23). The DMN plays an important role in emotion formation and processing, self-referential processing, and emotional appraisal (24, 25). Previous studies identified aberrant functional connectivity within and between DMN nodes (26–29) in patients with bipolar or unipolar depression. This finding might prove that a correlation exists between the DMN and the pathogenesis of depression. As for the medication-related changes in the DMN, some studies have been conducted on individuals with major depressive disorder (30, 31) and healthy people (32), which revealed that antidepressants changed the functional connectivity (30) and network flexibility (31) of the DMN. However, the role of the DMN in the treatment effects of bipolar II depression remains unclear.

Some issues arising from the above discussion need to be addressed. First, the fALFF change across different bands within the DMN when patients with bipolar II depression achieve remission through medicine therapy remains unclear. Second, in case that some fALFF changes in the DMN after treatment appear, the relation between these changes and depressive symptoms is unknown. Third, changes that occur in the different frequency bands of fALFF within the DMN are unknown.

Therefore, we carried out a longitudinal fMRI research on medication-free patients with bipolar II depression to understand fALFF changes across different frequencies after a 16-week treatment course with escitalopram and lithium.

We hypothesized that (1) treatment with escitalopram and lithium affects the fALFF in the DMN in bipolar II depression patients, (2) a correlation exists between the change in fALFF in the DMN and depression symptoms in bipolar II depression, and (3) fALFF changes across different bands within the DMN are different.

## Materials and Methods

### Subjects

Our research was conducted in line with the Declaration of Helsinki and approved by the Ethics Committee of the Second Xiangya Hospital of Central South University (approval number: 2019/004). Before the research, we explained the purpose and procedure of the study to all the participants or the participants' legal guardian and notified them that they could opt out any time.

Bipolar II disorder was diagnosed by two senior psychiatrists (Chen X.G, Tang J.S). Patients who met the following criteria were selected: (1) met the DSM-V (33) criteria for bipolar II disorder, were currently depressed, and scored >17 in the 17-item Hamilton Depression Rating Scale (HDRS) (34) and <7 in the Young Mania Rating Scale (YMRS) (35); (2) medication-free; and (3) between 16 and 40 years of age. We set the age criteria mainly because of the high incidence in the range (36) and to exclude brain aging as a confounding factor (37). HCs without history of psychiatric or neurological disease were recruited from the local community through advertisements and matched with patients in terms of age, education, and gender. All the participants were Han Chinese and right-handed. The exclusion criteria for all the subjects were as follows: (1) severe head trauma with loss of consciousness lasting more than 5 min, (2) any neurological disorder or other chronic somatic diseases, (3) alcohol or drug abuse, (4) history of a psychiatric disease or receiving antipsychotic treatments, and (5) any contraindication to MRI.

All the participants underwent functional MRI scans at baseline. The patients then received a 16-week treatment course that consisted of administration of escitalopram (12.50 ± 4.20 mg, daily) and lithium (793.48 ± 144.05 mg, daily). Treatment regimen and duration were based on CANMAT guidelines for the management of patients with bipolar disorder (2013) (38), a previous study (39), and our clinical experiences with bipolar II depression. After the 16-week treatment course, the patients were scheduled for follow-up fMRI scans. Patients with YMRS and HDRS scores of ≤ 7 were classified as remission status.

Twenty-eight medication-free patients with bipolar II depression were recruited from the outpatient clinic of the Department of Psychiatry at Second Xiangya Hospital, China.

One patient's fMRI data at baseline were excluded because of excessive head movement during the MRI scan; three patients' escitalopram with lithium treatment was transformed. Among these three, two patients presented with persistent side effects, such as nausea, sedation, and vomiting, and the other patient had no significant response. One patient refused to perform follow-up MRI scan because of discomfort during the test. Finally, 52 participants were included, 23 of whom were medication-free patients with bipolar II depression and 29 were HCs.

### Clinical and Neuropsychological Measurements

All bipolar II depression patients (*n* = 23) were evaluated at baseline and followed up with 17-item HDRS and YMRS. To ensure consistency and reliability of the ratings, two psychiatrists with over 5 years of clinical practice received a training session on how to use the HDRS and YMRS before the study was conducted. This procedure ensured that a correlation coefficient >0.8 was maintained over repeated assessments across the study.

### Image Acquisition

All MRI data were acquired using a 3T scanner (Siemens, Skyra, Germany) in Hunan Children's Hospital. Before the scan, we instructed all the subjects to stop moving and thinking of anything and to relax with eyes closed but not to fall asleep throughout the scan. Foam paddings were wrapped around the head to reduce head motion, and earplugs were used to attenuate scanner noise.

Sequence parameters were as follows: 36 slices; repetition time (TR)/echo time (TE) = 2,000/30 ms; flip angle (FA) = 90°; voxel size = 3.4 × 3.4 × 3.4 mm^3^; field of view (FOV) = 256 × 256 mm; and slice thickness = 3.4 mm. Each functional run consisted of 250 volumes and lasted for ~508 s. High-resolution T1-weighted three-dimensional structural images were acquired using a high-resolution sequence: 192 slices; TR/TE = 2,530/2.33 ms; voxel size = 1 × 1 × 1 mm^3^; FOV = 256 × 256 mm; flip angle (FA) = 7°; and slice thickness = 1 mm.

### Data Pre-processing

Data pre-processing was conducted with Data Processing Assistant for Resting-State fMRI (DPARSF, http://www.restfmri.net) (40), which is based on Statistical Parametric Mapping (SPM, https://www.fil.ion.ucl.ac.uk/spm/). The first 10 time points were removed for the reduction of the non-equilibrium effects of magnetization. The remaining 240 scans of each participant underwent slice timing, realignment, co-registration with the participants' own structural images, and segmentation. Then, the resulting images were normalized spatially with the standard Montreal Neurological Institute (MNI) EPI template in DARTEL and resampled to 3 × 3 × 3 mm^3^. Any participant with head motion of >1.5 mm translation or >1.5° rotation in any direction and mean FD Jenkinson of >0.2 mm was removed (41). No significant differences between the patients with bipolar II depression and HC were observed at baseline (*t* = −0.626, *p* = 0.534) and before and after treatment (*t* = 0.502, *p* = 0.621) in mean framewise displacement (FD Jenkinson). For the reduction of physiological noises, such as heartbeat and respirations, the signals of the white matter and cerebrospinal fluid and the 24 parameters of head motion (42) were regressed from the data. Furthermore, we performed scrubbing procedure (43) to eliminate the distance-dependent artifact of head motion. Adopting a 2–3-voxel FWHM in the smoothing process can produce objective results (44). Thus, we used a 6-mm Gaussian kernel to smoothen the generated images. Finally, detrending was performed for the elimination of the linear drift. We also performed the regression of global signals analysis to process the data (the detailed results shown in the Supplementary Material).

### Calculation of fALFF

fALFF analysis was performed with the DPARSF software. We computed fALFF values based on the method of previous studies (7, 18). The previous study demonstrated that the full fALFF frequency range (0.01–0.25 Hz) encompasses four bands: slow 5 (0.01–0.027 Hz), slow 4 (0.027–0.073 Hz), slow 3 (0.073–0.198 Hz), and slow 2 (0.198–0.25 Hz) (45). The fALFF of the slow 5 and slow 4 bands were calculated in our study. Firstly, we calculated the fALFF values of the slow 5, and we set the bands with 0.01–0.027 Hz; then, we set the bands with 0.027–0.073 in the DPARSF to calculate the slow 4 fALFF values.

### Statistical Analysis

The fALFF maps were estimated within the regions of the DMN template built in the GIFT toolbox (46). By subtracting the mean from the value of each voxel of the raw fALFF and then dividing by the standard deviation, we obtain a *Z*-standardized fALFF map of participants for the following statistical analysis. Voxel-wise independent two-sample *t*-test and paired *t*-tests in SPM8 were employed to compare the difference of fALFF between the patients and controls as well as before and after treatment in bipolar II depression patients, respectively. Age, gender, and educational years were considered covariates. Family-wise error (FWE) correction was utilized for multiple comparisons with a significance threshold of <0.025 and cluster size (CZ) of 100.

To examine the associations between the fALFF values and depressive symptoms, fALFF values in the baseline and follow-up patient groups were acquired from abnormal brain regions. Firstly, we picked every abnormal cluster as images, respectively; then, the fALFF values of every subject were calculated with “ROI signal of extractor” of DPARSF with this image as masks. In addition, the longitudinal changes in fALFF values (follow-up value minus baseline values) and decreased HDRS (baseline HDRS scores minus follow-up HDRS scores) were identified as changes in brain activity and clinical symptoms, respectively. Spearman correlation analysis was used in computing the correlation between the fALFF values and HDRS scores at baseline and between the changes in fALFF values and decreased HDRS scores.

Given that two comparisons (patients vs. controls at baseline, baseline patients vs. after-treatment patients) and four brain regions were included, for correlation analysis, the Bonferroni correction *p* < 0.006 (four regions, two stages, *p* < 0.05/4 × 2).

Clinical and demographic data were performed with SPSS (version 17.0). Mann–Whitney *U*-test was used in comparing differences between the patients and HCs in terms of age, marital status, and years of education. Differences in terms of gender and tobacco use were assessed using chi-square tests. In addition, to analyze the patients' longitudinal changes of HDRS, the Wilcoxon test was used. These statistical tests were two-tailed, and a *p-*value of < 0.05 was considered statistically significant.

## Results

### Demographic and Clinical Characteristics

A total of 52 participants were included, comprising 23 medication-free patients with bipolar II depression and 29 HCs. The demographic and clinical characteristics of the patients and HCs are shown in Table 1. The two groups did not differ significantly in terms of gender (*p* = 0.829), age (*p* = 0.934), tobacco use (*p* = 0.764), marital status (*p* = 0.577), and years of education (*p* = 0.118).

**Table 1:** Demographic and clinical characteristics of medication-free patients with bipolar II depression and HCs.

**Variables**	**Bipolar II depression (*N* = 23)**	**HCs (*N* = 29)**	**χ^**2**^/*Z***	***p*-value**
Gender—male/female, case	12/11	13/16	0.046	0.829^a^
Married (married/living as married)/non-married (widowed, divorced, separated, single), case	8/15	8/21	0.312	0.577^a^
Age—median (range), years	19 (16–39)	21 (15–32)	−0.083	0.934^b^
Education duration—median (range), years	14 (9–19)	14 (9–16)	−1.562	0.118^b^
Tobacco use—case	4	6	0.090	0.764^a^
Duration of illness—median (range), months	12 (1–96)	/	/	
Number of depressive episodes—median (range), times	2 (0–8)	/	/	
Number of hypomania episodes—median (range), times	1 (1–8)	/	/	
Age of onset—median (range), years	16 (10–38)	/	/	

HCs, healthy controls.

a The p-values for sex distribution, marital status, and tobacco use were obtained by chi-square-tests.

b *The p-value was obtained by the Mann–Whitney U-test applied*.

### Baseline and Longitudinal Changes in fALFF

**Figure 1:**
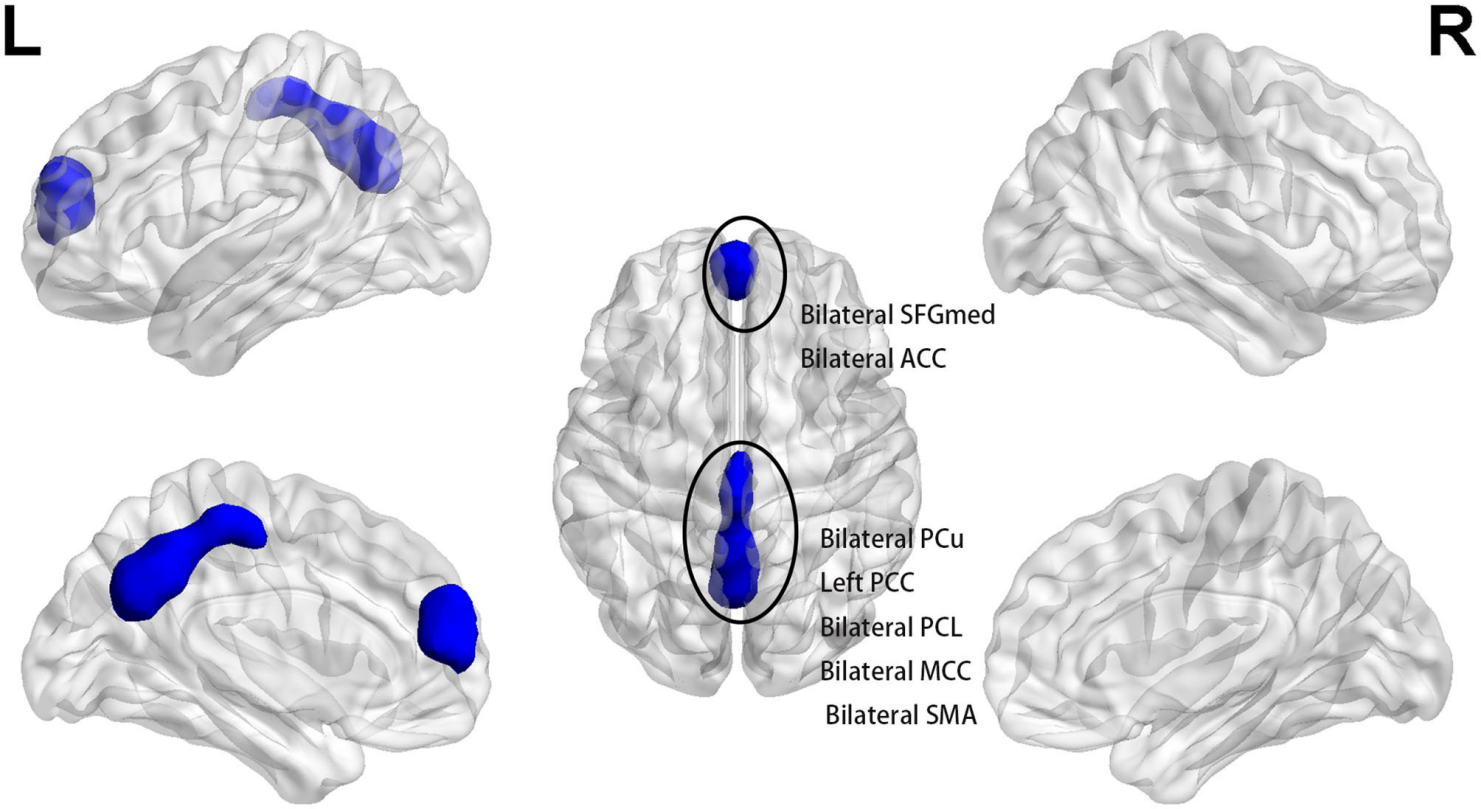
At baseline, patients exhibited lower slow 5 fALFF in bilateral SFGmed and bilateral ACC (cluster A) and bilateral PCu, left PCC, bilateral PCL, bilateral MCC, and bilateral SMA (cluster B) when compared with healthy controls (*p* < 0.025, FWE corrected, CZ = 100). SFGmed, medial superior frontal gyrus; ACC, anterior cingulate; PCu, precuneus; PCC, posterior cingulate; PCL, paracentral lobule; MCC, middle cingulum; SMA, supplementary motor area.

**Figure 2:**
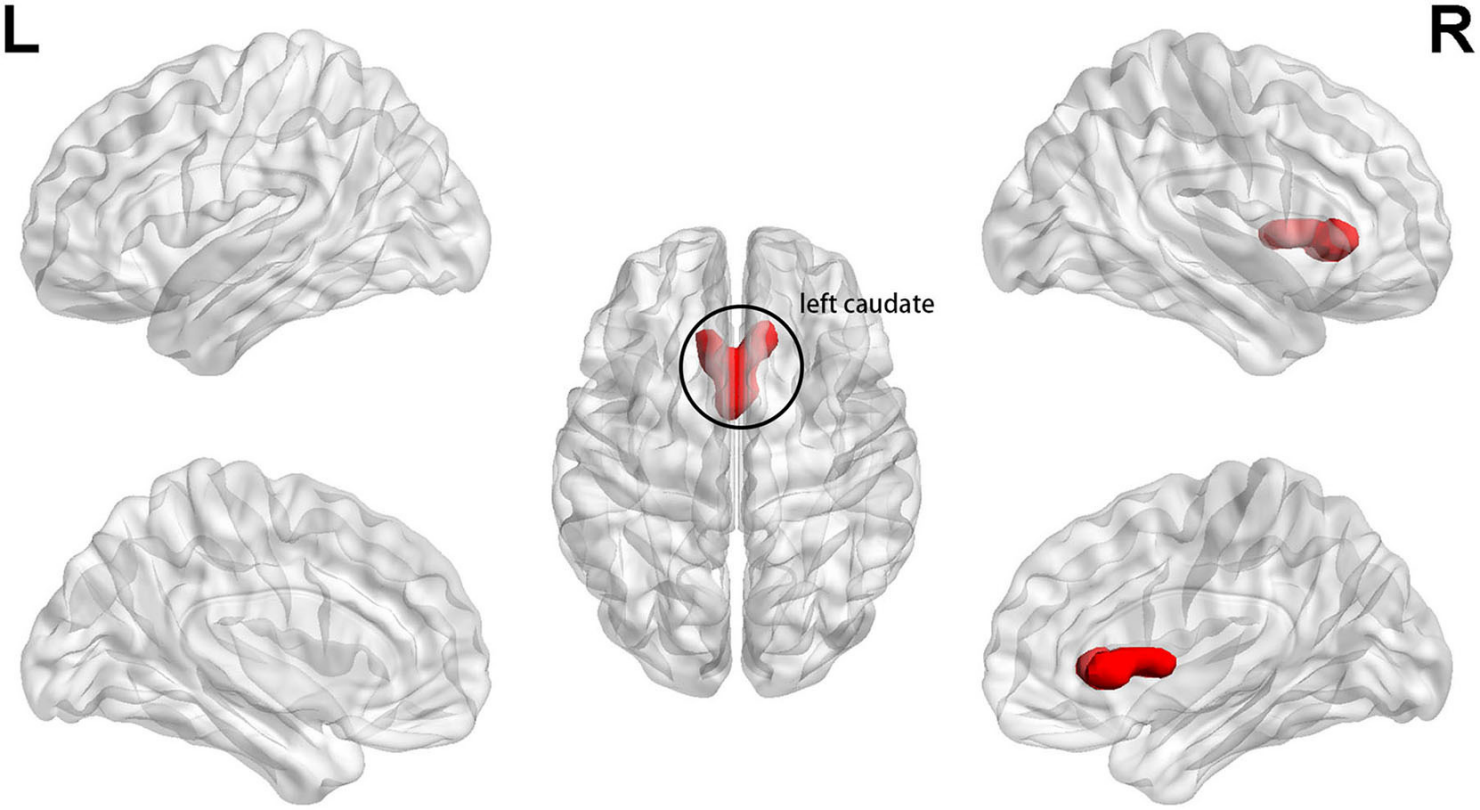
At baseline, patients exhibited higher slow 5 fALFF in left caudate (cluster C) when compared with healthy controls (*p* < 0.025, FWE corrected, CZ = 100).

At baseline, the patients displayed significantly lower fALFF values in clusters A and B than those of HCs in slow 5 band (*p* < 0.025, FWE corrected, CZ = 100). Cluster A included the bilateral medial SFG and bilateral anterior cingulate (ACC), and cluster B included bilateral PCu, left PCC, bilateral paracentral lobule, bilateral middle cingulum, and bilateral supplementary motor area (*p* < 0.025, FWE corrected, CZ = 100) (Figure 1 and Table 2). We also found that the patients had higher slow 5 fALFF values in cluster C (left caudate) than the HCs (*p* < 0.025, FWE corrected, CZ = 100) (Figure 2 and Table 2). However, no significant difference between the patients and HCs in the slow 4 band was observed (*p* < 0.025, FWE corrected, CZ = 100; Table 2).

**Table 2:** Baseline and longitudinal alterations of fALFF in bipolar II depression patients.

**Cluster**	**Brain region**	**Brodmann area**	**MNI coordinates**			**Voxels**	**Peak *T*-value**	***p* (FWE corrected)**
			***X***	***Y***	***Z***			
**Patients at baseline vs. controls**								
**0.01–0.027 band (slow 5 band)**								
Cluster A	Bilateral medial superior frontal gyrus Bilateral anterior cingulate	9/10/32	−3	48	15	181	−8.8003	<0.025
Cluster B	Bilateral precuneus Left posterior cingulate Bilateral paracentral lobule Bilateral middle cingulum Bilateral supplementary motor area	5/6/7/31	0	−54	45	536	−10.4495	<0.025
Cluster C	Left caudate	24	−3	15	6	128	9.3419	<0.025
0.027–0.073 band (slow 4 band)								
None								
**Patients at follow-up vs. baseline**								
**0.01–0.027 band (slow 5 band)**								
Cluster D	Bilateral medial superior frontal gyrus Bilateral superior frontal gyrus Bilateral anterior cingulate Right middle frontal gyrus	9/10	24	54	24	344	17.0949	<0.025
Cluster E	Bilateral precuneus Bilateral posterior cingulate Left cuneus	7/31	0	−66	33	181	13.0121	<0.025
Cluster F	Left angular Left middle temporal gyrus Left superior temporal gyrus Left supramarginal gyrus	39/40	−42	66	21	138	11.1745	<0.025
**0.027–0.073 band (slow 4 band)**								
None								
**Patients at follow-up vs. HC**								
**0.01–0.027 band (slow 5 band)**								
None								
**0.027–0.073 (slow 4 band)**								
None								

**Figure 3:**
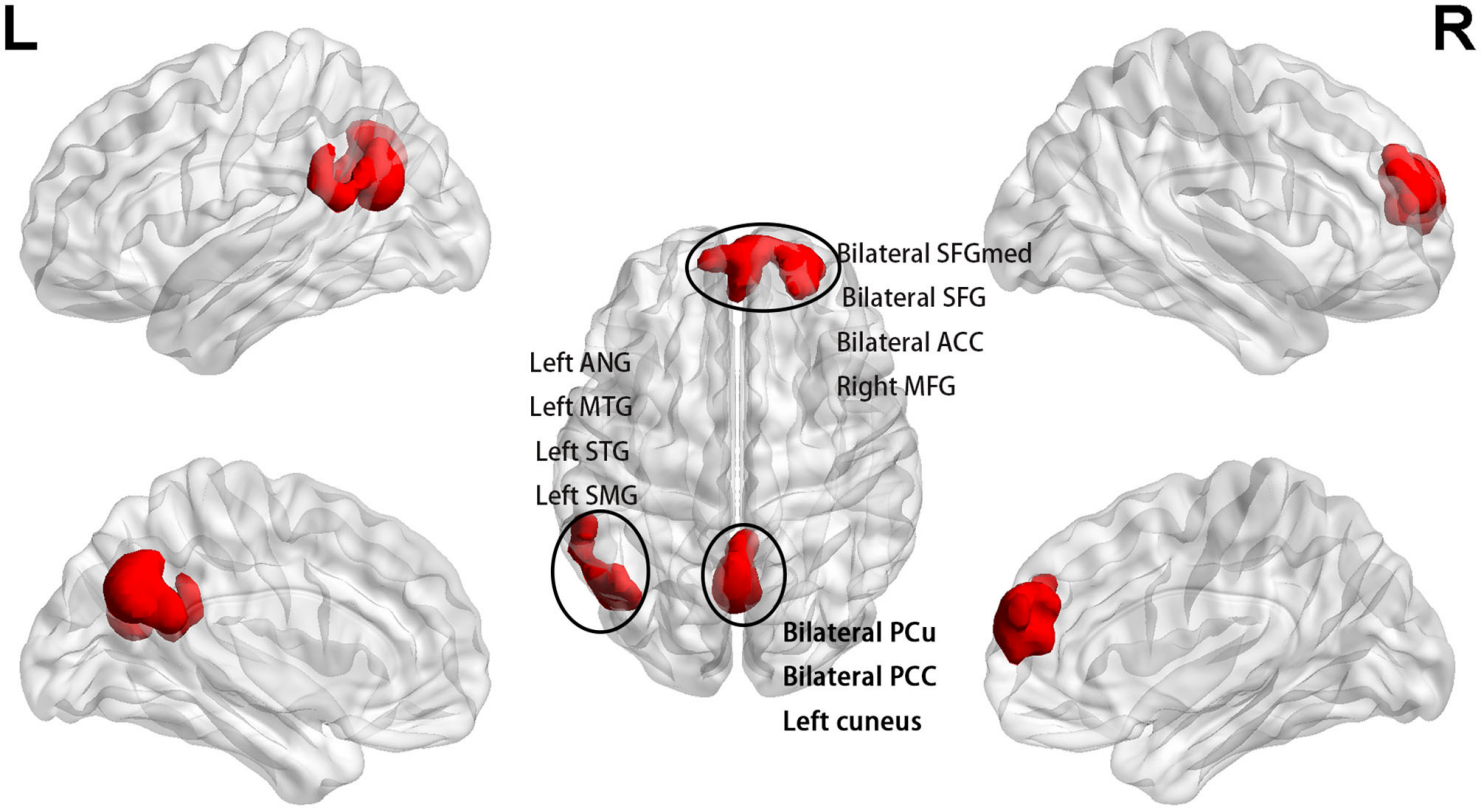
After treatment, fALFF increased in bilateral SFGmed, bilateral SFG, bilateral ACC, and right MFG (cluster D); bilateral PCu, bilateral PCC, and left cuneus (cluster E); and left ANG, left MTG, left STG, and left SMG (cluster F) in comparison with the baseline of the patients in the slow 5 band (*p* < 0.025, FWE corrected, CZ = 100). SFG, superior frontal gyrus; MFG, middle frontal gyrus; ANG, angular; MTG, middle temporal gyrus; STG, superior temporal gyrus; SMG, supramarginal gyrus.

After the 16-week treatment, fALFF increased in clusters D, E, and F compared with the baseline in the slow 5 band (*p* < 0.025, FWE corrected, CZ = 100; Figure 3 and Table 2). Cluster D included the bilateral medial SFG, bilateral SFG, bilateral ACC, and right middle frontal gyrus, and cluster E included the bilateral PCu, bilateral PCC, and left cuneus. Cluster F included the left angular gyrus, left middle temporal gyrus (MTG), left superior temporal gyrus (STG), and left supramarginal gyrus (SMG). None of the regions demonstrated decreased fALFF after treatment. There was no significant difference in slow 4 fALFF in the patients after treatment (*p* < 0.025, FWE corrected, CZ = 100; Table 2).

After the 16-week treatment, no significant differences between the patients and HC were observed (*p* < 0.025, FWE corrected, CZ = 100) in the slow 5 and slow 4 bands (Table 2).

### Longitudinal Alterations of Clinical Symptoms

The median (range) of the HDRS is 23 (29, 19) at baseline. After the 16-week treatment course, the HDRS was reduced to 3 (6, 0). HDRS decreased to 21 (26, 13). The Wilcoxon test revealed that the HDRS scores of patients were significantly higher than those of patients in the medication-free phase (*Z* = −4.202, *p* = 0.000). The median (range) of YMRS in the patients was 3 (4, 0) and 2 (3, 0) at baseline and follow-up, respectively.

### Relationships of Baseline and Longitudinal Alterations of fALFF and Clinical Variables

#### Relationships of Baseline and Longitudinal Alterations of fALFF and HDRS Scores

**Figure 4:**
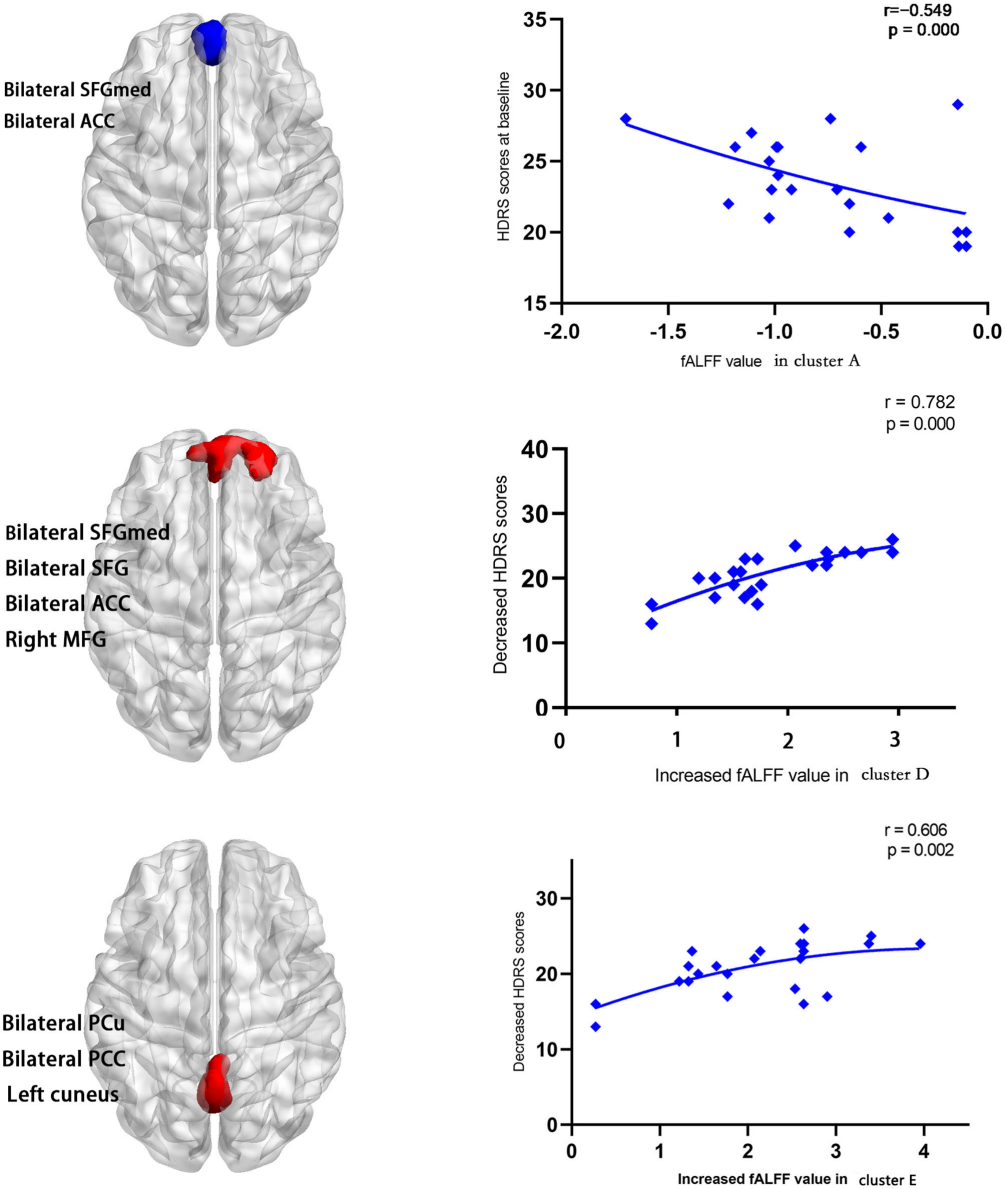
The baseline HDRS scores were negatively correlated with the slow 5 fALFF in bilateral SFGmed and bilateral ACC (cluster A) (*r* = −0.549, *p* = 0.000) at baseline. Decrease in HDRS scores (baseline HDRS scores minus follow-up HDRS scores) was positively correlated with the increased values in slow 5 fALFF (follow-up minus baseline) in bilateral SFGmed, bilateral SFG, bilateral ACC, and right MFG (cluster D) (*r* = 0.782, *p* = 0.000) and bilateral PCu, bilateral PCC, and left cuneus (cluster E) (*r* = 0.606, *p* = 0.002) at follow-up.

The baseline HDRS score was negatively correlated with the slow 5 fALFF of cluster A (*r* = −0.549, *p* = 0.000) at baseline (Figure 4). No correlation between the baseline of HDRS and cluster B (*r* = −0.509, *p* = 0.013) as well as cluster C (*r* = 0.094, *p* = 0.669) was found. The decrease in HDRS scores (baseline HDRS scores minus follow-up HDRS scores) was positively correlated with the increased values in slow 5 fALFF (follow-up minus baseline) in cluster D (*r* = 0.782, *p* = 0.000) and cluster E (*r* = 0.606, *p* = 0.002) at follow-up (Figure 4). No correlation between the decreases in HDRS scores and the increased values in slow 5 fALFF was found (*r* = 0.311, *p* = 0.149) in cluster F.

#### Relationships of Baseline fALFF and Other Clinical Variables

No correlation between the duration of illness and the slow 5 fALFF values in cluster A (*r* = −0.293, *p* = 0.174), cluster B (*r* = 0.259, *p* = 0.233), and cluster C (*r* = −0.090, *p* = 0.683) was found, respectively. Also, there was no correlation between the age of onset and the slow 5 fALFF values in cluster A (*r* = 0.142, *p* = 0.517), cluster B (*r* = −0.137, *p* = 0.534), and cluster C (*r* = 0.159, *p* = 0.470) respectively.

## Discussion

In the study, we explore treatment-related changes in fALFF within the DMN in patients with bipolar II depression who received a 16-week treatment course of escitalopram and lithium. The results confirm our hypothesis that treatment-related change in fALFF in the DMN can be corrected by escitalopram with lithium in patients with bipolar II depression. A correlation was observed between the change in fALFF in the DMN and depression symptoms in patients with bipolar II depression. fALFF changes across different bands within the DMN were different.

First, we will discuss the possible reasons for the increase in fALFF and reduced depressive symptoms after the treatment of bipolar II depression with escitalopram and lithium. Escitalopram is a selective serotonin reuptake inhibitor (SSRI) that increases serotonergic activity in the central nervous system by inhibiting 5-HT reuptake (47). Previous studies showed that serotonin (5-HT) has a role in long-term memory regulation (48, 49) and emotional regulation (50), and this role might be related to the pathogenesis of depression. The effective treatment of SSRI is related to the increase in circulation in 5-HT (51) and the concentration of 5-hydroxytryptamine in the serotonergic system including the prefrontal cortical area (52). A previous study has suggested that serotonergic effects on neurophysiology mainly occur at synaptic connection sites (53). When patients receive escitalopram treatment, changes in synaptic connections may be a cause of changes in spontaneous brain activity (54). As in our findings, one study reported escitalopram increased brain activities in the mPFC, LTC, and cuneus (55). Another research demonstrated the presence of a relationship between serotonin transporter occupancy and DMN connectivity (56). Serotonergic neurotransmitter system might play a role in modulating the spontaneous activity of the DMN and alleviating depression during escitalopram treatment.

Lithium is another medication used in our treatment program; it is effective in treating bipolar depression (57, 58). The several mechanisms of lithium, such as neurotransmitters, neuronal plasticity, and energy metabolism, possibly play a role in its therapeutic effects (59). Lithium may function in the prefrontal (60) and cingulate cortices (61) by regulating synaptic activity and energy metabolism. Synaptic activity and energy metabolism might be related to change in spontaneous brain activity. However, Vargas et al. (62) found that lithium has no effect on brain activation in euthymic patients with type I bipolar disorder. The reason for this difference may include the state of disease, study design, and sample size (only including 10 cases taking lithium). Given the inconsistent result, the change in the DMN by lithium in bipolar II depression needs further confirmation.

In our study, changes in fALFF after treatment with lithium and escitalopram in the DMN mainly occurred in the mPFC (bilateral medial SFG, bilateral ACC), PCU/PCC, left LTC (left MTG, left STG), and left IPL (left angular, left SMG) in bipolar II depression. In the subsequent texts, we discuss the possible role of these brain regions in the pathogenesis and treatment effect of bipolar II depression.

The first altered area was the mPFC, which is the core region of the DMN, after treatment of lithium and escitalopram. The slow 5 fALFF in these regions increased and were positively correlated with the decrease in HDRS. By using task-related and resting-state functional neuroimaging methods, some scientists reported that the mPFC is strongly related to self-referential thought, emotion regulation (63), maintaining spontaneous optimistic self-evaluative tendencies (64), and cognitive flexibility (65). Abnormal resting-state brain function in the mPFC was observed in individuals with bipolar disorder. One study reported reduced DMN connectivity and activation in the vmPFC in bipolar disorder patients with acute manic episodes (66). Another research showed decreased connectivity between the left crus II and bilateral mPFC in unmedicated patients with bipolar II depression (67). A recent fALFF study revealed fALFF values of bilateral superior frontal gyrus changed in bipolar depression (68). The mPFC is an area with strong serotonergic projections (69), and the SSRI increases the concentration of 5-hydroxytryptamine by increasing the excitability of interneurons in the PFC (52) and regulates the bold signal of mPFC (69). Chakroborty et al. (70) reported that escitalopram changed the spontaneous activity of the brain of rats. Evidence showed that escitalopram can increase sensitivity to positive words and decrease responses to self-referential words in the mPFC (50, 55). Lithium also exerts an effect on the mPFC. It modulates synaptic plasticity on PFC (60, 71) and increases the level of N-acetylaspartate in PFC, which participates in neuronal metabolism (72). Patients with bipolar disorder taking lithium showed increased spontaneous brain activity (73) and increased concentration of glutamate and glutamine (61) on ACC. Combining our findings and the findings mentioned above, we found that escitalopram and lithium might have an effect on the fALFF of the mPFC and depressive symptoms by modulating neurotransmitters, neuronal metabolism, and synaptic plasticity in bipolar II depression.

PCC/PCu is another important area of change in slow 5 fALFF within the DMN after treatment with escitalopram and lithium in patients with bipolar II depression. Slow 5 fALFF in these regions increased and were positively correlated with the decrease in HDRS. PCC/PCu plays an important role in making inferences regarding the mental states (74) and perceiving and processing psychosocial stress (75). Some scientists reported decreased fALFF of PCu in psychotic bipolar disorder by means of fMRI (21). Consistent with our results, some studies showed that escitalopram and lithium act on the cingulate gyrus: escitalopram can reduce posterior cingulate activity in healthy people (55), and lithium increased relative glucose metabolic rates of the posterior cingulate (76). Machado-Vieira et al. (77) reported that lithium achieves therapeutic effects by reducing lactate concentrations in the cingulate cortex in patients with bipolar I or II depression. Based on the above discussion, we explain why slow 5 fALFF in the cingulate cortex during bipolar depressive episodes can be corrected by escitalopram and lithium and why the depressive symptoms such as lower self-evaluation and reduced ability to cope with stress were alleviated.

We found that the lower fALFF value in the left IPL and left LTC can be corrected by escitalopram and lithium. Structural and functional abnormalities in the IPL and LTC in patients with bipolar disorder have been observed. Compared with the HC group, the bipolar depression group showed decreased fALFF in the left IPL (12), and the bipolar I disorder group displayed large temporal lobe white matter (78) and small volumes of STG (79). Both lithium and escitalopram have effects on the superior temporal gyrus. Escitalopram enhanced regional homogeneity in the superior temporal lobe in patients with panic disorder (80), and lithium treatment can increase superior temporal gyrus volume in bipolar I disorder (79). However, in the present study, we did not find a significant correlation between increased slow 5 fALFF in the left IPL and left LTC and a decrease in HDRS. The possible causes of these results were that the functions of these brain regions were mainly involved in cognition, auditory, and language function: the IPL is mainly related to cognitive functions, including memory retrieval and bottom-up attention (81); the MTG mainly takes part in motor skill learning (82) and short-term verbal memory (83); and the STG is generally considered a part of the high-order auditory cortex (84).

Finally, our study suggested that slow 5 fALFF better embodied the low-frequency amplitude of the cortex (i.e., mPFC, PCC) than slow 4 fALFF in patients with bipolar II depression. These findings are consistent with some previous studies (20, 45). Zuo found that the slow 4 fALFF showed the strongest signal in the basal ganglia thalamus, but slow 5 had the strongest signal in the cortex, particularly the mPFC (15). A resting-state study in post-stroke depression showed that fALFF only decreased in slow 5 band, but did not change in the slow 4 band (20): the changed brain region was observed in the cortex, including the right precentral gyrus and supplemental motor/middle frontal cortex. Another study in MDD suggested that alterations of slow 5 fALFF are more obvious than the slow 4 fALFF in cortical areas, such as the mPFC, ITG, and IPL (45). The neural mechanisms of the different frequency bands have not been elucidated yet, and some researchers proposed that neuronal oscillations are related to the activity of chemical synaptic signaling, input selection plasticity of neuron, and the connected neuron networks (85), which differ in the cortex and subcortex, and the information mentioned above may shed some light on our result that the cortex and subcortex have different fALFF bands.

## Limitation

The present study has some potential limitations. First, the sample size of our study is relatively small. Studies with larger sample sizes are needed to confirm these results in the future. Second, we adopted the combination of therapeutic regimens of escitalopram and lithium in line with the CANMAT guidelines for the management of patients with bipolar disorder (2013) and our clinical experience instead of the 2018 edition, which suggested the use of quetiapine as first-line treatment for medication-naive patients with bipolar II depression. In the future, monotherapy with quetiapine can also be used to prove the results. Third, the impact of comorbidity of patients was not taken into account in our study, which could be considered a limitation. Lastly, the HCs only had MRI at baseline, and thus, the effect of time on the brain may be biased as a confounding factor.

## Conclusions

Three main findings were obtained in this study. First, the hypoactivity of slow 5 fALFF in the DMN might be corrected by escitalopram with lithium. Second, a positive correlation between increased fALFF values and the improvement of depressive symptoms in the mPFC and PCu/PCC was found during follow-up. Finally, the experiment confirmed that the fALFF changes in patients with bipolar II depression are more sensitive to slow 5 band and the slow 5 fALFF might better embody the signal of the cortex when compared with slow 4 band.

## Data Availability Statement

The raw data supporting the conclusions of this article will be made available by the authors, without undue reservation.

## Ethics Statement

All the adult participants (≥18 years) signed informed consent forms. For participants under 18 years of age, written informed consent forms were provided by the participants' legal guardians/next of kin.

## Author Contributions

All authors listed have made a substantial, direct and intellectual contribution to the work, and approved it for publication.

## Conflict of Interest

The authors declare that the research was conducted in the absence of any commercial or financial relationships that could be construed as a potential conflict of interest.
