# Supplementary material for: Frequency-Specific Changes in the Fractional Amplitude of the Low-Frequency Fluctuations in the Default Mode Network in Medication-Free Patients With Bipolar II Depression: A Longitudinal Functional MRI Study
Source: Front Psychiatry. 2021 Jan 8;11:574819. doi: 10.3389/fpsyt.2020.574819 (PMC7819893; doi:10.3389/fpsyt.2020.574819)
Supplement: Supplementary file 1 [file Data_Sheet_1.doc]

Supplementary Information for

**Frequency-specific changes in the fractional amplitude of the low-frequency fluctuations in default mode network in medication-free patients with** **bipolar II depression: A longitudinal fMRI study**

**Jun Zhou^1^, Xiaoqian Ma^1^ , Chunwang Li ^2^, Aijun Liao^1^ , Zihao Yang^1^，Honghong Ren^1^, Jinsong Tang^3^, jinguang Li^1^, Zongchang Li^1^,Ying He^1⁎^ , Xiaogang Chen^1⁎^**

^1^Department of Psychiatry, The Second Xiangya Hospital, Central South University, Changsha, Hunan, China;National Clinical Research Center for Mental Disorders, Changsha, Hunan, China; National Technology Institute on Mental Disorders, Changsha, Hunan, China;Hunan Key Laboratory of Psychiatry and Mental Health, Changsha, Hunan, China;Mental Health Institute of Central South University, Changsha, Hunan, China

^2^ Department of Radiology, Hunan Children's Hospital, Changsha, China

^3^ Department of Psychiatry,Sir Run Run Shaw Hospital, Zhejiang University，Hangzhou,China

*** Correspondence:**

Ying He: [yinghe@csu.edu.cn](mailto:yinghe@csu.edu.cn)

Xiaogang Chen: chenxiaogang@csu.edu.cn

**Supplementary analysis with the regression of global signals in Data preprocessing**

**1 Data preprocessing**

Data preprocessing was conducted with Data Processing Assistant for Resting-State fMRI (DPARSF, http://www. restfmri. net)(1), which is based on Statistical Parametric Mapping (SPM, https://www. fil. ion. ucl. ac. uk/spm/). The first 10 time points were removed for the reduction of the nonequilibrium effects of magnetization. The remaining 240 scans of each participant underwent slice timing, realignment, co-registration with the participants’ own structural images, and segmentation. Then, the resulting images were normalized spatially with the standard Montreal Neurological Institute (MNI) EPI template in DARTEL and resampled to 3 × 3 × 3 mm3. Any participant with head motion of >1.5 mm translation or >1. 5° rotation in any direction and mean FD Jenkinson of >0. 2 mm were removed （2）. No significant differences between the patients with bipolar II depression and HC was observed at baseline (t = −0.626, *p* = 0. 534) and before and after treatment (t = 0.502, *p* = 0. 621) in mean framewise displacement (FD Jenkinson). For the reduction of physiological noises, such as heart beat and respirations, the signals of the white matter and cerebrospinal fluid, **global signals** and the 24 parameters of head motion(3) were regressed from the data. Furthermore, we performed scrubbing procedure (4) to eliminate the distance-dependent artifact of head motion. Adopting a 2–3-voxel FWHM in the smoothing process can produce objective results(5). Thus, we used a 6 mm Gaussian kernel to smoothen the generated images. Finally, Detrending was performed for the elimination of the linear drift.

**2 Calculation of fALFF**

fALFF analysis was performed with DPARSF software. We computed fALFF values based on the method of previous studies(6,7). The previous study demonstrated that the full fALFF frequency range (0. 01–0. 25 Hz) encompasses four bands: slow 5 (0.01–0. 027 Hz), slow 4 (0. 027–0. 073 Hz), slow 3 (0.073–0.198 Hz), and slow 2 (0.198–0.25 Hz) (8). The fALFF of the slow 5 and the slow 4 bands were calculated in our study.

1. **Statistical analysis**

The fALFF maps were estimated within the regions of DMN template built-in in GIFT toolbox(9), By subtracting the mean from the value of each voxel of the raw fALFF and then dividing by the standard deviation, we obtain Z-standardized fALFF map of participants for the following statistical analysis. Voxel-wise independent two-sample t-test and paired t-tests in SPM8 were employed to compare the difference of fALFF between the patients and controls as well as before and after treatment in bipolar II depression patients respectively. Age, gender, and educational years were considered covariates. Family-wise Error (FWE) correction was utilized for multiple comparisons with a significance threshold of <0. 025 and cluster size (CZ) of 100.

**4 Results**

At baseline, patients exhibited higher slow 5 fALFF in cluster A (Bilateral medial superior frontal gyrus) and cluster B (Bilateral Precuneus, bilateral post cingulate, bilateral middle cingulate ,left Cuneus) when compared with healthy controls (p < 0. 025, FWE corrected, CZ = 100, Supplementary Table1, Supplementary Figure 1). After treatment, fALFF decreased in clusters C, D, and E in comparison with the baseline of the patients in the slow 5 band (p < 0. 025, FWE corrected, CZ = 100, Supplementary Table1, Supplementary Figure 2). Cluster C included Bilateral medial superior frontal gyrus, bilateral superior frontal gyrus, bilateral middle frontal gyrus, left medial orbitofrontal gyrus; cluster D included bilateral precuneus, bilateral middle cingulate, bilateral post cingulate, left cuneus. Cluster E included left angular, left middle temporal gyrus, left inferior parietal lobe.

No significant difference in slow4 fALFF in the patients after treatment (*p* < 0. 025, FWE corrected, CZ = 100; Table 2).

**Supplementary Table 1 Baseline and Longitudinal changes of fALFF in bipolar II depression patients**

|  | Brain region | Brodmann area | MNI coordinates | | | Voxels | Peak T value | P (FWE corrected) |
| --- | --- | --- | --- | --- | --- | --- | --- | --- |
|  |  |  | X | Y | Z |  |  |  |
| Patients at baseline vs Controls | | | | | | | |  |
| 0. 01-0. 027 band (slow5 band) | | | | | | | |  |
| Cluster A | Bilateral medial superior frontal gyrus | 9/10 | -6 | 54 | 12 | 143 | 7.8644 | < 0.025 |
| Cluster B | Bilateral Precuneus  Bilateral post cingulate  Bilateral middle cingulate_  Left Cuneus | 7/31 | 3 | -57 | 42 | 301 | 8.3072 | < 0.025 |
| 0. 027-0. 073 band (slow4 band) | | | | | | | |  |
| none |  |  |  |  |  |  |  |  |
| **Patients at follow-up *vs* Baseline** | | | | | | | |  |
| **0. 01-0. 027 band (slow5 band)** | | | | | | | |  |
| Cluster C | Bilateral medial superior frontal gyrus  Bilateral superior frontal gyrus  Bilateral middle frontal gyrus  Left medial orbitofrontal gyrus | 9/10 | 6 | 63 | 18 | 239 | -11.5371 | < 0.025 |
| Cluster D | Bilateral precuneus  Bilateral middle cingulate  Bilateral post cingulate  Left cuneus | 7/31/23 | 3 | -45 | 33 | 390 | -18.4055 | < 0.025 |
| Cluster E | Left angular  Left middle temporal gyrus  Left inferior parietal lobe | 39/40 | 48 | -66 | 33 | 160 | -11.4631 | < 0.025 |
| **0. 027-0. 073 band (slow4 band)** | | | | | | | |  |
| none |  |  |  |  |  |  |  |  |
| **Patients at follow-up *vs HC*** | | | | | | | |  |
| **0. 01-0. 027 band (slow5 band)** | | | | | | | | |
| none |  |  |  |  |  |  |  |  |
| **0. 027-0. 073 (slow4 band)** | | | | | | | | |
| none |  |  |  |  |  |  |  |  |


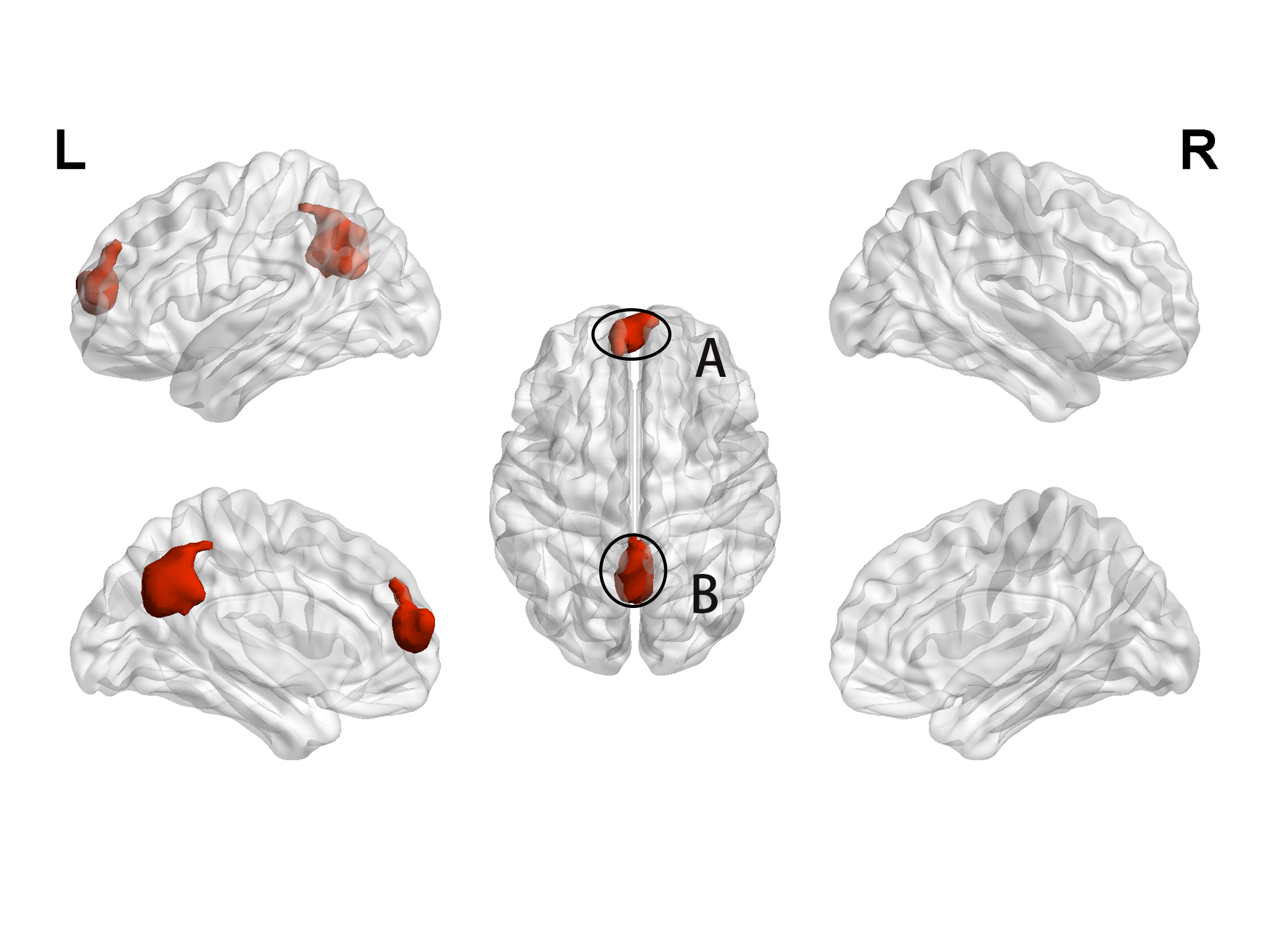


**Supplementary Figure1** At baseline, patients exhibited higher slow 5 fALFF in cluster A (Bilateral medial superior frontal gyrus) and cluster B (Bilateral Precuneus, bilateral post cingulate, bilateral middle cingulate ,left Cuneus) when compared with healthy controls (p < 0. 025, FWE corrected, CZ = 100).


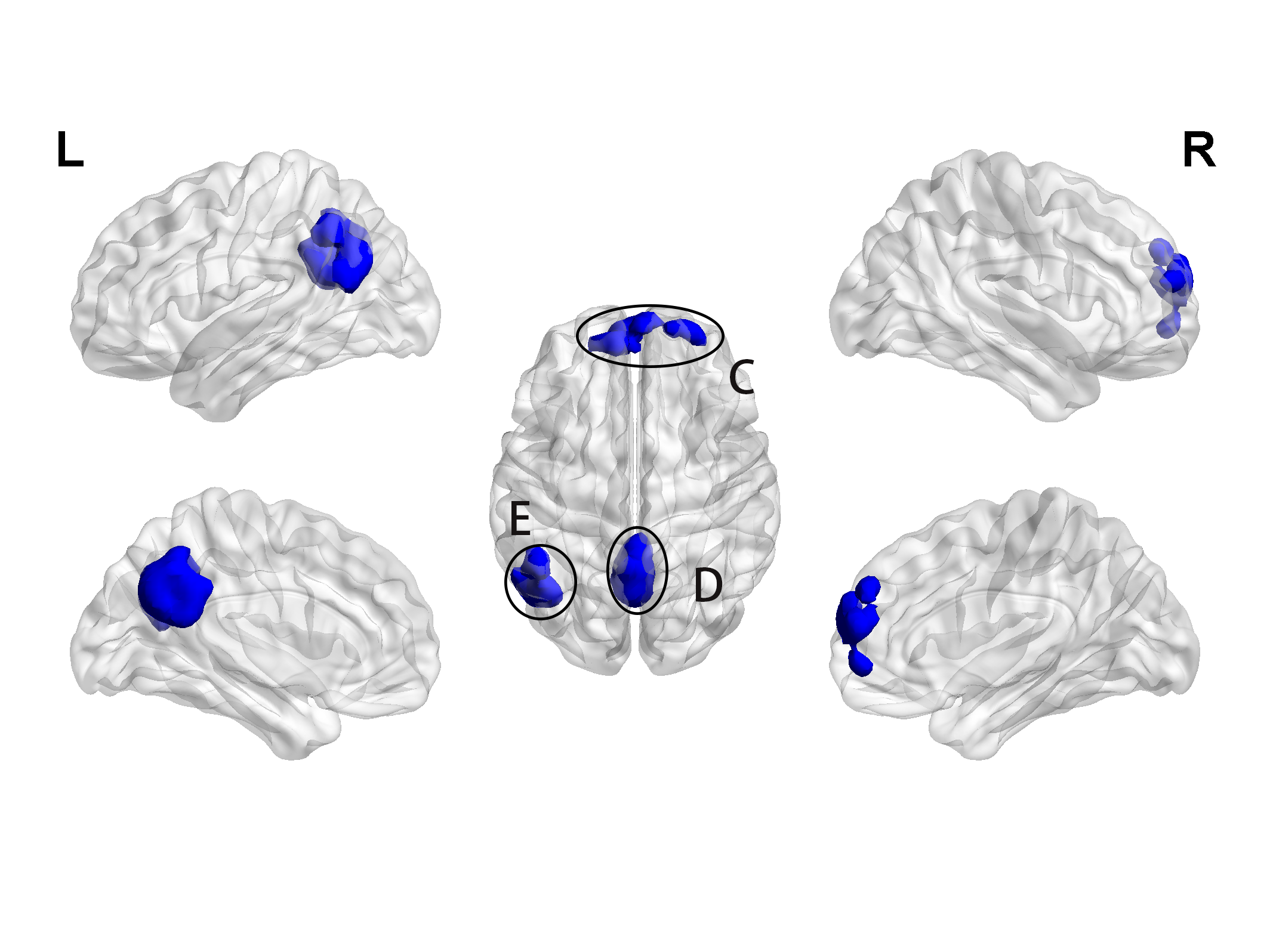


**Supplementary Figure2** After treatment, fALFF decreased in clusters C, D, and E in comparison with the baseline of the patients in the slow 5 band (p < 0. 025, FWE corrected, CZ = 100). Cluster C included Bilateral medial superior frontal gyrus, bilateral superior frontal gyrus, bilateral middle frontal gyrus, left medial orbitofrontal gyrus; cluster D included bilateral precuneus, bilateral middle cingulate, bilateral post cingulate, left cuneus. Cluster E included left angular, left middle temporal gyrus, left inferior parietal lobe.

**4 Discussion**

Some previous studies reported with and without global signal correction will produce different results in functional connectivity analysis. With global signal correction will cause the negative correlation, or anticorrelation (10). The degree of negative correlation is related to the size of the brain networks (11) and the brain regions(12). Scientists reported when global regression is performed, the global signal will be subtracted from the voxel’s time series, which induced negative correlation (11). We also found the different results with and without global signal correction. Without global signal correction, the longitudinal change of solw5 fALFF in bipolar II depression patients was positive, but when conducted the global signal regression, the longitudinal change of slow 5 fALFF was negative.

Reference

1 Yan C G, Wang XD, Zuo XN, Zang Y F. DPABI: Data Processing & Analysis for (Resting-State) Brain Imaging. Neuro informatics (2016) 14:339–351. doi: 10.1007/s12021-016-9299-4 (2016).

2 Yan CG, Craddock RC, Zuo XN, Zang YF, Milham MP. Standardizing the intrinsic brain: towards robust measurement of inter-individual variation in 1000 functional connectomes. Neuroimage( 2013)80:246-262. doi:10.1016/j.neuroimage.2013.04.081

3 Friston KJ, Williams S, Howard R, Frackowiak RS, Turner R. Movement-related effects in fMRI time-series. Magn Reson Med. (1996)35(3):346-355. doi:10.1002/mrm.1910350312.

4 Power JD, Mitra A, Laumann TO, Snyder AZ, Schlaggar BL, Petersen SE. Methods to detect, characterize, and remove motion artifact in resting state fMRI. Neuroimage. (2014)84:320-341. doi:10.1016/j.neuroimage.2013.08.048

5 Chen Z, Calhoun V. Effect of Spatial Smoothing on Task fMRI ICA and Functional Connectivity. Front Neuro sci. (2018)12:15. doi:10.3389/fnins.2018.00015

6 Li Y, Jing B, Liu H,  [Li](https://pubmed.ncbi.nlm.nih.gov/?term=Li+Y&cauthor_id=28550250) Y, [Gao](https://pubmed.ncbi.nlm.nih.gov/?term=Gao+X&cauthor_id=28550250) X, [Li](https://pubmed.ncbi.nlm.nih.gov/?term=Li+Y&cauthor_id=28550250) Y et al. Frequency-Dependent Changes in the Amplitude of Low-Frequency Fluctuations in Mild Cognitive Impairment with Mild Depression. J Alzheimers Dis. (2017)(58):1175‐1187. doi:10.3233/JAD-161282.

7 Zou QH, Zhu CZ, Yang Y, Zuo XN, Long XY, Cao QJ, et al. An improved approach to detection of amplitude of low-frequency fluctuation (ALFF) for resting-state fMRI: fractional ALFF. J Neurosci Methods (2008) 172:137–141.. doi: 10.1016/j.jneumeth.2008.04.012.

8 Wang L, Kong Q, Li K, Sua Y, Zeng Y, Zhang Q,et al. Frequency-dependent changes in amplitude of low-frequency oscillations in depression: A resting-state fMRI study. Neuro sci Lett (2016) 614:105‐111. doi:10.1016/j.neulet.2016.01.012

9 Calhoun V. D., Adali T., Pearlson G. D., Pekar J. J. A method for making group inferences from functional MRI data using independent component analysis. Human Brain Mapping. (2001)14(3):140–151. doi: 10.1002/hbm.1048.

10 Murphy K, Birn RM, Handwerker DA, Jones TB, Bandettini PA. The impact of global signal regression on resting state correlations: are anti-correlated networks introduced? Neuroimage. 2009;44(3):893-905. doi:10.1016/j.neuroimage.2008.09.036 11 Anderson JS, Druzgal TJ, Lopez-Larson M, Jeong EK, Desai K, Yurgelun-Todd D. Network anticorrelations, global regression, and phase-shifted soft tissue correction. Hum Brain Mapp. 2011;32(6):919-934. doi:10.1002/hbm.21079

12 Hayasaka S. Functional connectivity networks with and without global signal correction. Front Hum Neurosci. 2013;7:880. Published 2013 Dec 18. doi:10.3389/fnhum.2013.00880

**Supplementary analysis of Baseline and Longitudinal changes of ALFF in bipolar II depression patients**

**1 Data preprocessing**

Data preprocessing was conducted with Data Processing Assistant for Resting-State fMRI (DPARSF, http://www. restfmri. net)(1), which is based on Statistical Parametric Mapping (SPM, https://www. fil. ion. ucl. ac. uk/spm/). The first 10 time points were removed for the reduction of the nonequilibrium effects of magnetization. The remaining 240 scans of each participant underwent slice timing, realignment, co-registration with the participants’ own structural images, and segmentation. Then, the resulting images were normalized spatially with the standard Montreal Neurological Institute (MNI) EPI template in DARTEL and resampled to 3 × 3 × 3 mm3. Any participant with head motion of >1.5 mm translation or >1. 5° rotation in any direction and mean FD Jenkinson of >0. 2 mm were removed （2）. No significant differences between the patients with bipolar II depression and HC was observed at baseline (t = −0.626, *p* = 0. 534) and before and after treatment (t = 0.502, *p* = 0. 621) in mean framewise displacement (FD Jenkinson). For the reduction of physiological noises, such as heart beat and respirations, the signals of the white matter and cerebrospinal fluid and the 24 parameters of head motion(3) were regressed from the data. Furthermore, we performed scrubbing procedure (4) to eliminate the distance-dependent artifact of head motion. Adopting a 2–3-voxel FWHM in the smoothing process can produce objective results(5). Thus, we used a 6 mm Gaussian kernel to smoothen the generated images. Finally, Detrending was performed for the elimination of the linear drift. We also performed the regression of global signals analysis to process the data (The detailed results showed in supplementary information).

**2 Calculation of ALFF**

ALFF analysis was performed with DPARSF software. We computed ALFF values based on the method of previous studies(6,7). The previous study demonstrated that the full ALFF frequency range (0. 01–0. 25 Hz) encompasses four bands: slow 5 (0.01–0. 027 Hz), slow 4 (0. 027–0. 073 Hz), slow 3 (0.073–0.198 Hz), and slow 2 (0.198–0.25 Hz) (8). The ALFF of the slow 5 and the slow 4 bands were calculated in our study.

- 1. **Statistical analysis**

The ALFF maps were estimated within the regions of DMN template built-in in GIFT toolbox(9), By subtracting the mean from the value of each voxel of the raw ALFF and then dividing by the standard deviation, we obtain Z-standardized ALFF map of participants for the following statistical analysis. Voxel-wise independent two-sample t-test and paired t-tests in SPM8 were employed to compare the difference of ALFF between the patients and controls as well as before and after treatment in bipolar II depression patients respectively. Age, gender, and educational years were considered covariates. Family-wise Error (FWE) correction was utilized for multiple comparisons with a significance threshold of <0. 025 and cluster size (CZ) of 100.

**3 Result**

There is no significant difference between and within the group in slow 4 ALFF and slow 5 ALFF (p< 0.025, FWE corrected, CZ=100).

**4 Discussion**

Previous studies also reported that different results in ALFF and fALFF, which demonstrated that fALFF reduced prominently in the ventricle and cistern areas, while increasing in the PCC and MPFC when compared with that of ALFF(10), and the brain areas within the default mode network have significantly higher fALFF than the rest of the brain(11,12) which was consistent with our study. Because of the small simple size of our study, we will enlarge the sample size to further study the difference between ALFF and fALFF.

Reference

1 Yan C G, Wang XD, Zuo XN, Zang Y F. DPABI: Data Processing & Analysis for (Resting-State) Brain Imaging. Neuro informatics (2016) 14:339–351. doi: 10.1007/s12021-016-9299-4 (2016).

2 Yan CG, Craddock RC, Zuo XN, Zang YF, Milham MP. Standardizing the intrinsic brain: towards robust measurement of inter-individual variation in 1000 functional connectomes. Neuroimage( 2013)80:246-262. doi:10.1016/j.neuroimage.2013.04.081

3 Friston KJ, Williams S, Howard R, Frackowiak RS, Turner R. Movement-related effects in fMRI time-series. Magn Reson Med. (1996)35(3):346-355. doi:10.1002/mrm.1910350312.

4 Power JD, Mitra A, Laumann TO, Snyder AZ, Schlaggar BL, Petersen SE. Methods to detect, characterize, and remove motion artifact in resting state fMRI. Neuroimage. (2014)84:320-341. doi:10.1016/j.neuroimage.2013.08.048

5 Chen Z, Calhoun V. Effect of Spatial Smoothing on Task fMRI ICA and Functional Connectivity. Front Neuro sci. (2018)12:15. doi:10.3389/fnins.2018.00015

6 Liu CH, Li F, Li SF, et al. Abnormal baseline brain activity in bipolar depression: a resting state functional magnetic resonance imaging study. Psychiatry Res. 2012;203(2-3):175-179. doi:10.1016/j.pscychresns.2012.02.007

7 Zou QH, Zhu CZ, Yang Y, Zuo XN, Long XY, Cao QJ, et al. An improved approach to detection of amplitude of low-frequency fluctuation (ALFF) for resting-state fMRI: fractional ALFF. J Neurosci Methods (2008) 172:137–141.. doi: 10.1016/j.jneumeth.2008.04.012.

8 Wang L, Kong Q, Li K, Sua Y, Zeng Y, Zhang Q,et al. Frequency-dependent changes in amplitude of low-frequency oscillations in depression: A resting-state fMRI study. Neuro sci Lett (2016) 614:105‐111. doi:10.1016/j.neulet.2016.01.012

9 Calhoun V. D., Adali T., Pearlson G. D., Pekar J. J. A method for making group inferences from functional MRI data using independent component analysis. Human Brain Mapping. (2001)14(3):140–151. doi: 10.1002/hbm.1048.

10 Zou QH, Zhu CZ, Yang Y, et al. An improved approach to detection of amplitude of low-frequency fluctuation (ALFF) for resting-state fMRI: fractional ALFF. J Neurosci Methods. 2008;172(1):137-141.

11 He Y, Zang YF, Jiang TZ, Liang M, Gong GL. Detecting functional connectivity of the cerebellum using low frequency fluctuations (LFFs). Medical image computing and computer-assisted intervention. Lect Notes Comput Sci. 2004; 3217:907–15.

12 Raichle ME, MacLeod AM, Snyder AZ, Powers WJ, Gusnard DA, Shulman GL. A default mode of brain function. Proc Natl Acad Sci USA. 2001; 98:676–82.
