# Supplementary material for: Frequency-Specific Changes in the Fractional Amplitude of the Low-Frequency Fluctuations in the Default Mode Network in Medication-Free Patients With Bipolar II Depression: A Longitudinal Functional MRI Study
Source: Front Psychiatry. 2021 Jan 8;11:574819. doi: 10.3389/fpsyt.2020.574819 (PMC7819893; doi:10.3389/fpsyt.2020.574819)
Supplement: Supplementary file 2 [file Data_Sheet_1.ZIP › WithoutGSR_ALFF/1_ALFF0.01_0.027/1_Cresult/1-C FWE 0.05.docx]

Only——Threshold

-- p value = 8.8133e-06

-- intensity = 4.78

-- cluster size = 5

Number of clusters found: 1

----------------------

Cluster 1

Number of voxels: 8

Peak MNI coordinate: 0 54 45

Peak MNI coordinate region: // undefined // undefined // undefined // undefined // undefined // undefined

Peak intensity: -5.4149

# voxels structure

8 --TOTAL # VOXELS--

2 Frontal_Sup_Medial_L (aal)

1 Frontal_Sup_Medial_R (aal)

D:\2019SCI\2020SCirevise\Revise_0.027ALFF\1_CTestICAmask\spmT_0001.nii,1

Type: T

df: 47

Threshold

-- p value = 8.8133e-06

-- intensity = 4.78

-- cluster size = 20

Number of clusters found: 2

----------------------

Cluster 1

Number of voxels: 88

Peak MNI coordinate: 42 -66 33

Peak MNI coordinate region: // Right Cerebrum // Parietal Lobe // Angular Gyrus // Gray Matter // brodmann area 39 // Angular_R (aal)

Peak intensity: 6.8639

# voxels structure

88 --TOTAL # VOXELS--

88 Right Cerebrum

73 White Matter

53 Angular_R (aal)

48 Parietal Lobe

40 Temporal Lobe

27 Superior Temporal Gyrus

25 Angular Gyrus

19 Temporal_Mid_R (aal)

18 Supramarginal Gyrus

15 Gray Matter

10 brodmann area 39

8 Middle Temporal Gyrus

7 Inferior Parietal Lobule

7 Parietal_Inf_R (aal)

5 brodmann area 40

4 Occipital_Mid_R (aal)

3 Temporal_Sup_R (aal)

2 Sub-Gyral

1 Precuneus

----------------------

Cluster 2

Number of voxels: 47

Peak MNI coordinate: 24 18 45

Peak MNI coordinate region: // Right Cerebrum // Frontal Lobe // Middle Frontal Gyrus // White Matter // undefined // Frontal_Sup_R (aal)

Peak intensity: 7.0877

# voxels structure

47 --TOTAL # VOXELS--

47 Frontal Lobe

47 Right Cerebrum

35 White Matter

28 Frontal_Sup_R (aal)

22 Middle Frontal Gyrus

19 Frontal_Mid_R (aal)

16 Superior Frontal Gyrus

12 Gray Matter

9 brodmann area 8

8 Sub-Gyral

3 brodmann area 6

1 Medial Frontal Gyrus

>>
