# Supplementary material for: Frequency-Specific Changes in the Fractional Amplitude of the Low-Frequency Fluctuations in the Default Mode Network in Medication-Free Patients With Bipolar II Depression: A Longitudinal Functional MRI Study
Source: Front Psychiatry. 2021 Jan 8;11:574819. doi: 10.3389/fpsyt.2020.574819 (PMC7819893; doi:10.3389/fpsyt.2020.574819)
Supplement: Supplementary file 2 [file Data_Sheet_1.ZIP › WithGSR_FALLFF/FALFF0.01_0.027_Gloab/1cResult/reportonly+fwe0.025.doc]

1c

>> xjview

D:\FALFF0.027_Gloab\1cResult\spmT_0001.img,1

Type: T

df: 48

Threshold

-- p value = 3.1745e-06

-- intensity = 5.07

-- cluster size = 100

Number of clusters found: 2

----------------------

Cluster 1

Number of voxels: 143

Peak MNI coordinate: -6 54 12

Peak MNI coordinate region: // Left Cerebrum // Frontal Lobe // Medial Frontal Gyrus // White Matter // undefined // Frontal_Sup_Medial_L (aal)

Peak intensity: 7.8644

\# voxels	structure

143	--TOTAL # VOXELS--

118	Frontal Lobe

96	Frontal_Sup_Medial_L (aal)

92	Medial Frontal Gyrus

81	Left Cerebrum

64	Gray Matter

53	White Matter

49	brodmann area 10

38	Right Cerebrum

34	Frontal_Sup_Medial_R (aal)

24	Inter-Hemispheric

23	Superior Frontal Gyrus

15	brodmann area 9

4	Cingulum_Ant_R (aal)

4	Cingulum_Ant_L (aal)

3	Frontal_Sup_L (aal)

2	Frontal_Sup_R (aal)

----------------------

Cluster 2

Number of voxels: 301

Peak MNI coordinate: 3 -57 42

Peak MNI coordinate region: // Right Cerebrum // Parietal Lobe // Precuneus // Gray Matter // brodmann area 7 // Precuneus_R (aal)

Peak intensity: 8.3072

\# voxels	structure

301	--TOTAL # VOXELS--

194	Precuneus

191	Parietal Lobe

164	Left Cerebrum

137	Gray Matter

131	Precuneus_L (aal)

116	Right Cerebrum

93	Precuneus_R (aal)

77	brodmann area 7

65	Limbic Lobe

59	White Matter

48	Cingulate Gyrus

48	brodmann area 31

21	Inter-Hemispheric

21	Cingulum_Post_L (aal)

20	Posterior Cingulate

15	Cingulum_Post_R (aal)

15	Cuneus_L (aal)

14	Frontal Lobe

14	Paracentral Lobule

14	Cingulum_Mid_L (aal)

10	Occipital Lobe

8	Cingulum_Mid_R (aal)

5	brodmann area 23

5	brodmann area 5

4	Cuneus

3	Paracentral_Lobule_L (aal)

2	brodmann area 30

1	Calcarine_L (aal)

>>
