# Supplementary material for: Frequency-Specific Changes in the Fractional Amplitude of the Low-Frequency Fluctuations in the Default Mode Network in Medication-Free Patients With Bipolar II Depression: A Longitudinal Functional MRI Study
Source: Front Psychiatry. 2021 Jan 8;11:574819. doi: 10.3389/fpsyt.2020.574819 (PMC7819893; doi:10.3389/fpsyt.2020.574819)
Supplement: Supplementary file 2 [file Data_Sheet_1.ZIP › WithGSR_FALLFF/FALFF0.01_0.027_Gloab/2_1Result/report5.88only-.doc]

2-1only_

Toolbox Path Cache read in 0.04 seconds.

MATLAB Path initialized in 1.14 seconds.

>> xjview

D:\2019SCI\2020SCirevise_Brain\FALFF0.027_Gloab\12Result\spmT_0003.img,1

Type: T

df: 22

Threshold

-- p value = 3.2258e-06

-- intensity = 5.88

-- cluster size = 100

Number of clusters found: 3

----------------------

Cluster 1

Number of voxels: 239

Peak MNI coordinate: 6 63 18

Peak MNI coordinate region: // Right Cerebrum // Frontal Lobe // undefined // Gray Matter // brodmann area 10 // Frontal_Sup_Medial_R (aal)

Peak intensity: -11.5371

\# voxels	structure

239	--TOTAL # VOXELS--

211	Frontal Lobe

120	Left Cerebrum

115	White Matter

98	Medial Frontal Gyrus

97	Superior Frontal Gyrus

92	Right Cerebrum

91	Frontal_Sup_Medial_L (aal)

90	Gray Matter

67	brodmann area 10

42	Frontal_Sup_Medial_R (aal)

34	Frontal_Sup_R (aal)

26	Inter-Hemispheric

23	brodmann area 9

23	Frontal_Sup_L (aal)

19	Frontal_Med_Orb_L (aal)

15	Frontal_Mid_R (aal)

13	Middle Frontal Gyrus

9	Frontal_Mid_L (aal)

3	Cingulum_Ant_R (aal)

2	Cingulum_Ant_L (aal)

1	Frontal_Med_Orb_R (aal)

----------------------

Cluster 2

Number of voxels: 160

Peak MNI coordinate: -48 -63 24

Peak MNI coordinate region: // Left Cerebrum // Temporal Lobe // Middle Temporal Gyrus // Gray Matter // brodmann area 39 // Angular_L (aal)

Peak intensity: -11.4631

\# voxels	structure

160	--TOTAL # VOXELS--

160	Left Cerebrum

94	White Matter

79	Parietal Lobe

79	Temporal Lobe

72	Angular_L (aal)

61	Gray Matter

46	Middle Temporal Gyrus

39	Temporal_Mid_L (aal)

37	Inferior Parietal Lobule

37	Parietal_Inf_L (aal)

35	brodmann area 39

32	Superior Temporal Gyrus

29	Angular Gyrus

23	brodmann area 40

14	Supramarginal Gyrus

10	Occipital_Mid_L (aal)

2	brodmann area 22

2	Occipital Lobe

2	SupraMarginal_L (aal)

2	Precuneus

1	brodmann area 19

----------------------

Cluster 3

Number of voxels: 390

Peak MNI coordinate: 3 -45 33

Peak MNI coordinate region: // Right Cerebrum // Limbic Lobe // Cingulate Gyrus // Gray Matter // brodmann area 31 // Cingulum_Mid_R (aal)

Peak intensity: -18.4055

\# voxels	structure

390	--TOTAL # VOXELS--

225	Left Cerebrum

192	Precuneus

186	Parietal Lobe

164	Gray Matter

159	Precuneus_L (aal)

154	Limbic Lobe

138	Right Cerebrum

104	White Matter

99	Precuneus_R (aal)

81	Posterior Cingulate

75	Cingulate Gyrus

73	brodmann area 7

68	brodmann area 31

47	Cingulum_Post_L (aal)

27	Inter-Hemispheric

23	Cuneus_L (aal)

22	Cingulum_Post_R (aal)

17	Cingulum_Mid_R (aal)

17	Cingulum_Mid_L (aal)

16	Occipital Lobe

12	brodmann area 23

9	brodmann area 30

8	Frontal Lobe

8	Paracentral Lobule

6	Cuneus

1	Sub-Gyral

1	Calcarine_L (aal)

1	brodmann area 5

1	Paracentral_Lobule_L (aal)

1	brodmann area 29

>>
