# Supplementary material for: Frequency-Specific Changes in the Fractional Amplitude of the Low-Frequency Fluctuations in the Default Mode Network in Medication-Free Patients With Bipolar II Depression: A Longitudinal Functional MRI Study
Source: Front Psychiatry. 2021 Jan 8;11:574819. doi: 10.3389/fpsyt.2020.574819 (PMC7819893; doi:10.3389/fpsyt.2020.574819)
Supplement: Supplementary file 2 [file Data_Sheet_1.ZIP › WithoutGSR_ALFF/1_ALFF0.01_0.027/1_2result/12_FDR0.025C100.doc]

D:\2019SCI\2020SCirevise\Revise_0.027ALFF\1_2TestICAmask\spmT_0001.nii,1

Type: T

df: 22

Threshold

-- p value = 0.0017294

-- intensity = 3.2753

-- cluster size = 100

Number of clusters found: 1

----------------------

Cluster 1

Number of voxels: 207

Peak MNI coordinate: -9 30 42

Peak MNI coordinate region: // Left Cerebrum // Frontal Lobe // Medial Frontal Gyrus // White Matter // undefined // Frontal_Sup_Medial_L (aal)

Peak intensity: -7.1982

\# voxels	structure

207	--TOTAL # VOXELS--

207	Left Cerebrum

182	Frontal Lobe

146	White Matter

98	Medial Frontal Gyrus

85	Frontal_Sup_Medial_L (aal)

74	Frontal_Sup_L (aal)

67	Superior Frontal Gyrus

50	Gray Matter

25	Limbic Lobe

23	Frontal_Mid_L (aal)

18	Cingulate Gyrus

16	brodmann area 32

14	Anterior Cingulate

11	brodmann area 9

11	Cingulum_Ant_L (aal)

10	brodmann area 6

7	brodmann area 8

6	brodmann area 10

6	Middle Frontal Gyrus

5	Cingulum_Mid_L (aal)

4	Sub-Gyral

>>
