# Supplementary material for: Frequency-Specific Changes in the Fractional Amplitude of the Low-Frequency Fluctuations in the Default Mode Network in Medication-Free Patients With Bipolar II Depression: A Longitudinal Functional MRI Study
Source: Front Psychiatry. 2021 Jan 8;11:574819. doi: 10.3389/fpsyt.2020.574819 (PMC7819893; doi:10.3389/fpsyt.2020.574819)
Supplement: Supplementary file 2 [file Data_Sheet_1.ZIP › WithoutGSR_ALFF/1_ALFF0.01_0.027/1_2result/12FDR矫正次C20.doc]

xjview

D:\2019SCI\2020SCirevise\Revise_0.027ALFF\1_2TestICAmask\spmT_0001.nii,1

Type: T

df: 22

Threshold

-- p value = 5.3285e-05

-- intensity = 4.71

-- cluster size = 20

Number of clusters found: 1

----------------------

Cluster 1

Number of voxels: 24

Peak MNI coordinate: -9 30 42

Peak MNI coordinate region: // Left Cerebrum // Frontal Lobe // Medial Frontal Gyrus // White Matter // undefined // Frontal_Sup_Medial_L (aal)

Peak intensity: -7.1982

\# voxels	structure

24	--TOTAL # VOXELS--

24	Left Cerebrum

22	Frontal_Sup_Medial_L (aal)

22	Frontal Lobe

18	Medial Frontal Gyrus

14	White Matter

8	Gray Matter

6	Cingulate Gyrus

4	brodmann area 32

2	brodmann area 6

2	Limbic Lobe

1	Frontal_Sup_L (aal)

1	brodmann area 8

1	brodmann area 9

1	Cingulum_Mid_L (aal)

>>
