# Supplementary material for: Frequency-Specific Changes in the Fractional Amplitude of the Low-Frequency Fluctuations in the Default Mode Network in Medication-Free Patients With Bipolar II Depression: A Longitudinal Functional MRI Study
Source: Front Psychiatry. 2021 Jan 8;11:574819. doi: 10.3389/fpsyt.2020.574819 (PMC7819893; doi:10.3389/fpsyt.2020.574819)
Supplement: Supplementary file 2 [file Data_Sheet_1.ZIP › WithoutGSR_ALFF/1_ALFF0.01_0.027/1_Cresult/1-cFER0.025t2.72.doc]

FDR0.025

ONLY-__df: 22

Threshold

-- p value = 0.0040159

-- intensity = 2.7686

-- cluster size = 100

Number of clusters found: 1

----------------------

Cluster 1

Number of voxels: 122

Peak MNI coordinate: 0 54 45

Peak MNI coordinate region: // undefined // undefined // undefined // undefined // undefined // undefined

Peak intensity: -5.4149

\# voxels	structure

122	--TOTAL # VOXELS--

44	Frontal Lobe

35	Right Cerebrum

35	Gray Matter

32	Frontal_Sup_Medial_R (aal)

32	Superior Frontal Gyrus

32	Frontal_Sup_Medial_L (aal)

21	brodmann area 8

17	Frontal_Sup_R (aal)

14	brodmann area 9

13	Inter-Hemispheric

10	Medial Frontal Gyrus

9	Left Cerebrum

1	Frontal_Mid_R (aal)

1	Frontal_Sup_L (aal)

D:\2019SCI\2020SCirevise\Revise_0.027ALFF\1_CTestICAmask\spmT_0001.nii,1

Type: T

df: 47

Threshold

-- p value = 0.0040159

-- intensity = 2.7686

-- cluster size = 100

Number of clusters found: 4

Only+

Cluster 1

Number of voxels: 191

Peak MNI coordinate: -39 -57 30

Peak MNI coordinate region: // Left Cerebrum // Parietal Lobe // Angular Gyrus // White Matter // undefined // Angular_L (aal)

Peak intensity: 5.9701

\# voxels	structure

191	--TOTAL # VOXELS--

191	Left Cerebrum

147	White Matter

106	Temporal Lobe

92	Angular_L (aal)

85	Parietal Lobe

51	Superior Temporal Gyrus

49	Middle Temporal Gyrus

42	Temporal_Mid_L (aal)

40	Gray Matter

40	Supramarginal Gyrus

36	Angular Gyrus

33	brodmann area 39

20	Parietal_Inf_L (aal)

19	Occipital_Mid_L (aal)

11	Inferior Parietal Lobule

6	brodmann area 40

3	SupraMarginal_L (aal)

3	Precuneus

1	brodmann area 22

1	Sub-Gyral

----------------------

Cluster 2

Number of voxels: 309

Peak MNI coordinate: 42 -66 33

Peak MNI coordinate region: // Right Cerebrum // Parietal Lobe // Angular Gyrus // Gray Matter // brodmann area 39 // Angular_R (aal)

Peak intensity: 6.8639

\# voxels	structure

309	--TOTAL # VOXELS--

309	Right Cerebrum

249	White Matter

183	Parietal Lobe

163	Angular_R (aal)

125	Temporal Lobe

84	Supramarginal Gyrus

76	Superior Temporal Gyrus

57	Gray Matter

56	Inferior Parietal Lobule

46	Angular Gyrus

42	Temporal_Mid_R (aal)

29	brodmann area 39

27	Middle Temporal Gyrus

23	brodmann area 40

20	Temporal_Sup_R (aal)

19	Parietal_Inf_R (aal)

16	Sub-Gyral

11	SupraMarginal_R (aal)

8	Occipital_Mid_R (aal)

5	brodmann area 13

3	Precuneus

1	Sub-lobar

1	Insula

----------------------

Cluster 3

Number of voxels: 377

Peak MNI coordinate: 24 18 45

Peak MNI coordinate region: // Right Cerebrum // Frontal Lobe // Middle Frontal Gyrus // White Matter // undefined // Frontal_Sup_R (aal)

Peak intensity: 7.0877

\# voxels	structure

377	--TOTAL # VOXELS--

375	Frontal Lobe

374	Right Cerebrum

307	White Matter

202	Frontal_Sup_R (aal)

202	Superior Frontal Gyrus

114	Frontal_Mid_R (aal)

111	Middle Frontal Gyrus

66	Gray Matter

40	Sub-Gyral

40	brodmann area 8

40	Frontal_Sup_Medial_R (aal)

21	Medial Frontal Gyrus

14	brodmann area 6

13	Supp_Motor_Area_R (aal)

7	brodmann area 9

4	brodmann area 10

2	Inter-Hemispheric

2	Supp_Motor_Area_L (aal)

2	Precentral_R (aal)

1	Frontal_Sup_Medial_L (aal)

1	Left Cerebrum

1	Cingulate Gyrus

1	brodmann area 32

----------------------

Cluster 4

Number of voxels: 212

Peak MNI coordinate: -24 12 48

Peak MNI coordinate region: // Left Cerebrum // Frontal Lobe // Middle Frontal Gyrus // White Matter // undefined // Frontal_Mid_L (aal)

Peak intensity: 6.7586

\# voxels	structure

212	--TOTAL # VOXELS--

212	Frontal Lobe

212	Left Cerebrum

159	White Matter

122	Frontal_Sup_L (aal)

107	Superior Frontal Gyrus

85	Frontal_Mid_L (aal)

73	Middle Frontal Gyrus

51	Gray Matter

32	brodmann area 8

25	Sub-Gyral

11	brodmann area 9

8	brodmann area 6

7	Medial Frontal Gyrus

4	Frontal_Sup_Medial_L (aal)

1	Precentral_L (aal)
