# Supplementary material for: Frequency-Specific Changes in the Fractional Amplitude of the Low-Frequency Fluctuations in the Default Mode Network in Medication-Free Patients With Bipolar II Depression: A Longitudinal Functional MRI Study
Source: Front Psychiatry. 2021 Jan 8;11:574819. doi: 10.3389/fpsyt.2020.574819 (PMC7819893; doi:10.3389/fpsyt.2020.574819)
Supplement: Supplementary file 2 [file Data_Sheet_1.ZIP › WithoutGSR_FALFF/1_FALFF0.01_0.027/1_Cresult/reportFWE0.025t5.07.doc]

>> xjview

D:\Submitmap_revise\WithoutGSR_FALFF\1_FALFF0.01_0.027\1_Cresult\spmT_0001.nii,1

Type: T

df: 47

Threshold

-- p value = 3.3183e-06

-- intensity = 5.07

-- cluster size = 100

Number of clusters found: 1

----------------------

Cluster 1

Number of voxels: 128

Peak MNI coordinate: -3 15	6

Peak MNI coordinate region: // Left Cerebrum // Sub-lobar // Lateral Ventricle // Cerebro-Spinal Fluid // undefined // Caudate_L (aal)

Peak intensity: 9.3419

\# voxels	structure

128	--TOTAL # VOXELS--

101	Sub-lobar

64	White Matter

57	Left Cerebrum

55	Right Cerebrum

41	Extra-Nuclear

35	Gray Matter

35	Corpus Callosum

33	Caudate

33	Caudate Head

27	Lateral Ventricle

27	Cerebro-Spinal Fluid

23	Caudate_L (aal)

16	Inter-Hemispheric

16	Limbic Lobe

16	Anterior Cingulate

9	Sub-Gyral

9	Frontal Lobe

4	Caudate_R (aal)

3	Olfactory_R (aal)

2	brodmann area 24

2	Cingulum_Ant_R (aal)

1	Olfactory_L (aal)

D:\Submitmap_revise\WithoutGSR_FALFF\1_FALFF0.01_0.027\1_Cresult\spmT_0001.nii,1

Type: T

df: 47

Threshold

-- p value = 3.3183e-06

-- intensity = 5.07

-- cluster size = 100

Number of clusters found: 2

----------------------

Cluster 1

Number of voxels: 181

Peak MNI coordinate: -3 48 15

Peak MNI coordinate region: // Left Cerebrum // Frontal Lobe // Medial Frontal Gyrus // Gray Matter // brodmann area 10 // Cingulum_Ant_L (aal)

Peak intensity: -8.8003

\# voxels	structure

181	--TOTAL # VOXELS--

122	Frontal Lobe

112	Frontal_Sup_Medial_L (aal)

101	Medial Frontal Gyrus

92	Gray Matter

83	Left Cerebrum

49	Right Cerebrum

49	Inter-Hemispheric

46	brodmann area 10

38	brodmann area 9

38	Frontal_Sup_Medial_R (aal)

32	White Matter

21	Superior Frontal Gyrus

17	Cingulum_Ant_L (aal)

12	Cingulum_Ant_R (aal)

9	Limbic Lobe

9	Anterior Cingulate

8	brodmann area 32

2	Frontal_Med_Orb_L (aal)

----------------------

Cluster 2

Number of voxels: 536

Peak MNI coordinate: 0 -54 45

Peak MNI coordinate region: // Left Cerebrum // Parietal Lobe // Precuneus // undefined // undefined // Precuneus_L (aal)

Peak intensity: -10.8425

\# voxels	structure

536	--TOTAL # VOXELS--

331	Left Cerebrum

281	Precuneus

270	Parietal Lobe

240	Gray Matter

200	Precuneus_L (aal)

172	Right Cerebrum

143	Frontal Lobe

122	White Matter

108	Precuneus_R (aal)

100	brodmann area 7

82	Paracentral Lobule

75	brodmann area 31

69	Limbic Lobe

62	Medial Frontal Gyrus

54	Cingulate Gyrus

51	Cingulum_Mid_L (aal)

48	Paracentral_Lobule_L (aal)

46	brodmann area 6

33	Inter-Hemispheric

28	Cuneus_L (aal)

21	Occipital Lobe

20	Paracentral_Lobule_R (aal)

19	Cingulum_Mid_R (aal)

18	Supp_Motor_Area_R (aal)

17	brodmann area 5

15	Supp_Motor_Area_L (aal)

15	Posterior Cingulate

12	Cingulum_Post_L (aal)

8	Cuneus

7	Cingulum_Post_R (aal)

2	brodmann area 23

2	Calcarine_L (aal)

1	Sub-Gyral
