# Supplementary material for: Frequency-Specific Changes in the Fractional Amplitude of the Low-Frequency Fluctuations in the Default Mode Network in Medication-Free Patients With Bipolar II Depression: A Longitudinal Functional MRI Study
Source: Front Psychiatry. 2021 Jan 8;11:574819. doi: 10.3389/fpsyt.2020.574819 (PMC7819893; doi:10.3389/fpsyt.2020.574819)
Supplement: Supplementary file 2 [file Data_Sheet_1.ZIP › WithoutGSR_FALFF/1_FALFF0.01_0.027/2_1reult/FWE0.025T5.87.doc]

D:\Submitmap_revise\WithoutGSR_FALFF\1_FALFF0.01_0.027\2_1reult\spmT_0002.img,1

Type: T

df: 22

Threshold

-- p value = 3.3024e-06

-- intensity = 5.87

-- cluster size = 100

Number of clusters found: 3

----------------------

Cluster 1

Number of voxels: 344

Peak MNI coordinate: 24 54 24

Peak MNI coordinate region: // Right Cerebrum // Frontal Lobe // Middle Frontal Gyrus // White Matter // undefined // Frontal_Mid_R (aal)

Peak intensity: 17.0949

\# voxels	structure

344	--TOTAL # VOXELS--

293	Frontal Lobe

243	Right Cerebrum

150	Gray Matter

143	Superior Frontal Gyrus

137	White Matter

115	Medial Frontal Gyrus

101	Frontal_Sup_Medial_R (aal)

92	brodmann area 10

81	Frontal_Sup_R (aal)

76	Frontal_Sup_Medial_L (aal)

59	Left Cerebrum

50	brodmann area 9

44	Frontal_Mid_R (aal)

42	Inter-Hemispheric

32	Middle Frontal Gyrus

19	Cingulum_Ant_R (aal)

11	Frontal_Sup_L (aal)

10	Cingulum_Ant_L (aal)

8	Anterior Cingulate

8	Limbic Lobe

7	brodmann area 32

2	Frontal_Med_Orb_L (aal)

1	brodmann area 6

----------------------

Cluster 2

Number of voxels: 138

Peak MNI coordinate: -42 -66 21

Peak MNI coordinate region: // Left Cerebrum // Temporal Lobe // Middle Temporal Gyrus // White Matter // undefined // Temporal_Mid_L (aal)

Peak intensity: 11.1745

\# voxels	structure

138	--TOTAL # VOXELS--

138	Left Cerebrum

98	White Matter

84	Temporal Lobe

55	Angular_L (aal)

54	Parietal Lobe

43	Middle Temporal Gyrus

40	Temporal_Mid_L (aal)

38	Superior Temporal Gyrus

35	Gray Matter

25	brodmann area 39

22	Angular Gyrus

19	SupraMarginal_L (aal)

18	Inferior Parietal Lobule

17	Supramarginal Gyrus

13	Temporal_Sup_L (aal)

10	brodmann area 40

7	Occipital_Mid_L (aal)

4	Parietal_Inf_L (aal)

----------------------

Cluster 3

Number of voxels: 181

Peak MNI coordinate: 0 -66 33

Peak MNI coordinate region: // Left Cerebrum // Parietal Lobe // Precuneus // undefined // undefined // Precuneus_L (aal)

Peak intensity: 13.0121

\# voxels	structure

181	--TOTAL # VOXELS--

112	Precuneus

102	Parietal Lobe

93	Left Cerebrum

79	Precuneus_L (aal)

79	Gray Matter

71	Right Cerebrum

59	Precuneus_R (aal)

47	Limbic Lobe

45	brodmann area 7

33	White Matter

32	Posterior Cingulate

20	brodmann area 31

17	Inter-Hemispheric

15	Occipital Lobe

14	Cuneus_L (aal)

13	Cingulate Gyrus

13	Cingulum_Post_L (aal)

12	Cingulum_Post_R (aal)

9	brodmann area 23

7	Cuneus

5	brodmann area 30

1	Calcarine_L (aal)

>>
