# Supplementary material for: Frequency-Specific Changes in the Fractional Amplitude of the Low-Frequency Fluctuations in the Default Mode Network in Medication-Free Patients With Bipolar II Depression: A Longitudinal Functional MRI Study
Source: Front Psychiatry. 2021 Jan 8;11:574819. doi: 10.3389/fpsyt.2020.574819 (PMC7819893; doi:10.3389/fpsyt.2020.574819)
Supplement: Supplementary file 2 [file Data_Sheet_1.ZIP › WithoutGSR_FALFF/3_FALFF0.01_0.073_0.198/1_Cresult/reportFDR0.025T4.06C100无结果.doc]

Only-

D:\falff0.073_0.198\1_Cresult\spmT_0001.img,1

Type: T

df: 47

Threshold

-- p value = 9.2184e-05

-- intensity = 4.06

-- cluster size = 20

Number of clusters found: 1

----------------------

Cluster 1

Number of voxels: 23

Peak MNI coordinate: 45 -51 15

Peak MNI coordinate region: // Right Cerebrum // Temporal Lobe // Superior Temporal Gyrus // White Matter // undefined // Temporal_Mid_R (aal)

Peak intensity: -6.5804

\# voxels	structure

23	--TOTAL # VOXELS--

23	Temporal Lobe

23	Right Cerebrum

22	White Matter

21	Superior Temporal Gyrus

17	Temporal_Mid_R (aal)

3	Temporal_Sup_R (aal)

1	Angular_R (aal)

1	Gray Matter

1	Supramarginal Gyrus

1	brodmann area 40

1	Middle Temporal Gyrus

Only+

Cluster 1

Number of voxels: 25

Peak MNI coordinate: -9 24 36

Peak MNI coordinate region: // Left Cerebrum // Limbic Lobe // Cingulate Gyrus // Gray Matter // brodmann area 32 // Frontal_Sup_Medial_L (aal)

Peak intensity: 7.0415

\# voxels	structure

25	--TOTAL # VOXELS--

17	Left Cerebrum

17	Gray Matter

16	Frontal_Sup_Medial_L (aal)

16	Frontal Lobe

12	Cingulate Gyrus

9	Medial Frontal Gyrus

8	brodmann area 32

5	brodmann area 6

5	Limbic Lobe

4	Inter-Hemispheric

4	Cingulum_Mid_L (aal)

4	brodmann area 9

4	Right Cerebrum

2	Cingulum_Mid_R (aal)

2	Frontal_Sup_Medial_R (aal)

1	Supp_Motor_Area_L (aal)

>>
